# Cranioencephalic functional lymphoid units in glioblastoma

**DOI:** 10.1038/s41591-024-03152-x

**Published:** 2024-07-31

**Authors:** Celia Dobersalske, Laurèl Rauschenbach, Yichao Hua, Christoph Berliner, Anita Steinbach, Anika Grüneboom, Konstantinos D. Kokkaliaris, Dieter H. Heiland, Pia Berger, Sarah Langer, Chin L. Tan, Martin Stenzel, Somaya Landolsi, Flora Weber, Marvin Darkwah Oppong, Rudolf A. Werner, Hanah Gull, Thomas Schröder, Thomas Linsenmann, Andreas K. Buck, Matthias Gunzer, Martin Stuschke, Kathy Keyvani, Michael Forsting, Martin Glas, Jonathan Kipnis, Dennis A. Steindler, Hans Christian Reinhardt, Edward W. Green, Michael Platten, Alpaslan Tasdogan, Ken Herrmann, Florian Rambow, Igor Cima, Ulrich Sure, Björn Scheffler

**Affiliations:** 1 German Cancer Consortium (DKTK), partner site Essen/Düsseldorf, a partnership between DKFZ and University Hospital Essen, University Duisburg–Essen, Essen, Germany; 2 German Cancer Research Center (DKFZ), Heidelberg, Germany; 3 DKFZ Division Translational Neurooncology at the WTZ, University Medicine Essen, Essen, Germany; 4 West German Cancer Center (WTZ), University Hospital Essen, Essen, Germany; 5 Department of Neurosurgery and Spine Surgery, University Hospital Essen, Essen, Germany; 6 Center for Translational Neuroscience and Behavioral Science (C-TNBS), University of Duisburg–Essen, Essen, Germany; 7 Department of Applied Computational Cancer Research, IKIM, University Hospital Essen, Essen, Germany; 8 Department of Nuclear Medicine, University Hospital Essen, Essen, Germany; 9 Leibniz-Institut für Analytische Wissenschaften—ISAS—e.V., Dortmund, Germany; 10 Dr. Senckenberg Institute of Pathology, University Hospital Frankfurt, Frankfurt am Main, Germany; 11 DKTK, German Cancer Consortium, partner site Frankfurt/Mainz, Quantitative Spatial Cancer Biology Laboratory, University Hospital Frankfurt, Frankfurt am Main, Germany; 12 Frankfurt Cancer Institute (FCI), Goethe University Frankfurt, Frankfurt am Main, Germany; 13 DKTK, German Cancer Consortium, partner site Freiburg, Translational Neurosurgery, Microenvironment and Immunology Research Laboratory, University of Freiburg, Freiburg, Germany; 14 Department of Neurosurgery, University Clinic Erlangen, Erlangen, Germany; 15 Department of Neurological Surgery, Northwestern University Feinberg School of Medicine, Chicago, IL USA; 16 CCU Neuroimmunology and Brain Tumor Immunology, German Cancer Research Center, Heidelberg, Germany; 17 DKTK, German Cancer Consortium, Core Center Heidelberg, Heidelberg, Germany; 18 Department of Neurology, Medical Faculty Mannheim, Mannheim Center for Translational Neuroscience, Heidelberg University, Mannheim, Germany; 19 Department of Nuclear Medicine, University Hospital Würzburg, Würzburg, Germany; 20 University Hospital Frankfurt, Department of Nuclear Medicine, Clinic for Radiology and Nuclear Medicine, Frankfurt am Main, Germany; 21 The Russell H. Morgan Department of Radiology, Division of Nuclear Medicine and Molecular Imaging, Johns Hopkins University School of Medicine, Baltimore, MD USA; 22 Department of Hematology and Stem Cell Transplantation, University Hospital Essen, Essen, Germany; 23 Department of Neurosurgery, University Hospital Würzburg, Würzburg, Germany; 24 Institute for Experimental Immunology and Imaging, University Hospital, University Duisburg–Essen, Essen, Germany; 25 Department of Radiation Oncology, University Hospital Essen, Essen, Germany; 26 Institute of Neuropathology, University Hospital Essen, Essen, Germany; 27 Department of Diagnostic and Interventional Radiology and Neuroradiology, University Hospital Essen, Essen, Germany; 28 Department of Neurology, Division of Neurooncology, University Hospital Essen, Essen, Germany; 29 Brain Immunology and Glia (BIG) Center, Washington University School of Medicine in St Louis, St Louis, MO USA; 30 Department of Pathology and Immunology, Washington University School of Medicine in St Louis, St Louis, MO USA; 31 Steindler Consulting, Boston, MA USA; 32 The Eshelman Institute for Innovation, The University of North Carolina at Chapel Hill, Chapel Hill, NC USA; 33 Center of Medical Biotechnology (ZMB), University Duisburg–Essen, Essen, Germany; 34 Immune Monitoring Unit, National Center for Tumor Diseases, Heidelberg, Germany; 35 Helmholtz Institute for Translational Oncology, Mainz, Germany; 36 German Cancer Research Center-Hector Cancer Institute at the Medical Faculty Mannheim, University of Heidelberg, Mannheim, Germany; 37 Department of Dermatology, University Hospital Essen, Essen, Germany

**Keywords:** CNS cancer, Tumour immunology

## Abstract

The ecosystem of brain tumors is considered immunosuppressed, but our current knowledge may be incomplete. Here we analyzed clinical cell and tissue specimens derived from patients presenting with glioblastoma or nonmalignant intracranial disease to report that the cranial bone (CB) marrow, in juxtaposition to treatment-naive glioblastoma tumors, harbors active lymphoid populations at the time of initial diagnosis. Clinical and anatomical imaging, single-cell molecular and immune cell profiling and quantification of tumor reactivity identified CD8^+^ T cell clonotypes in the CB that were also found in the tumor. These were characterized by acute and durable antitumor response rooted in the entire T cell developmental spectrum. In contrast to distal bone marrow, the CB niche proximal to the tumor showed increased frequencies of tumor-reactive CD8^+^ effector types expressing the lymphoid egress marker S1PR1. In line with this, cranial enhancement of CXCR4 radiolabel may serve as a surrogate marker indicating focal association with improved progression-free survival. The data of this study advocate preservation and further exploitation of these cranioencephalic units for the clinical care of glioblastoma.

## Abstract (web summary)

Analyses of tumor and bone marrow tissue from patients with glioblastoma demonstrate the presence of extracerebral niches that contained tumor-reactive and memory T cell subsets, including early stem-like phenotypes and stages, indicating antitumor CD8^+^ T cell differentiation in cranial bone marrow.

## Main

The classic perception of the brain as an immune-privileged organ with very limited immune activity is outdated^1^. Recent research connects brain function and immunosurveillance to guardian immune cells assembling on the outer borders of the brain, which has changed the way we currently view brain immunity in health and disease. Evidence continues to arise on innate and adaptive immune cells residing within the choroid plexus, the meninges and the dural sinuses, together acting as neuro-immune interface^2–4^. These immune cells are strategically positioned to sense intracranial cues delivered via interstitial, cerebrospinal and lymphatic fluid drainage from the brain^1,5^. There are also direct connections between the brain and the meninges provided by bridging veins that cross the intermediary barrier, forming designated arachnoid cuff exit points^6^. This allows immune cell trafficking and hence dynamic and remote control over brain function. As a further extension, the meninges are connected with the overlying skull bone marrow by osseous channels in the calvaria, the superior part of the skull bone^1^^,^^7^. These channels contain blood vessels that link the meningeal circulation to the sinusoidal vasculature of the bone marrow. Hematopoietic stem and progenitor cells in the perisinusoidal niches generate erythroid cells, together with lineages of myelocytes and lymphocytes that can traffic through these channels into the underlying meninges^7^. It is therefore not surprising that various intracerebral circumstances foster local hematopoietic responses. This is, for example, evident by the altered egress of myeloid and B cells from the bone marrow of skull to the meningeal borders in animal models of brain injury, inflammation and aging^1,7^.

As our understanding of immunosurveillance in the brain continues to evolve, we can learn much from its disruption during malignant disease, for example, from glioblastoma, the most malignant adult brain cancer, that remains uniformly lethal with a median survival of less than 2 years^8^. Immune checkpoint-inhibiting immunotherapies have proven to be of limited effect in patients with glioblastoma. Several immunosuppressive resistance mechanisms are considered to be in place. These may involve systemic immunosuppression, including prevention of immune cell infiltration via the blood–brain barrier, sequestration of immune cells in the bone marrow or iatrogenic destruction of peripheral immune cells^9^. In addition, cycles of intrinsic, adaptive and acquired mechanisms of immunotherapy resistance are discussed on the basis of heterogeneous molecular subtypes of the tumor, the exhausted nature of infiltrating T cells and the tumor-promoting effect to reprogram myeloid cells in the microenvironment^9,10^. While these factors are extensively studied within the tumor parenchyma, the involvement of adjacent structures of the neuro-immune interface remains unknown. Specifically, the skull bone is less intensely studied in adult humans compared to animal models, and it is far more difficult to access than the marrow of routinely evaluated hip bone^11^. Because bone marrow sites shrink with increasing age in the human body^12^, substantial hematopoietic activity was per se not expected in the cranial bone (CB) of a disease that frequently affects advanced stages of life. In this study, we explored the immune cell repertoire within the marrow of the CB to assess their prevalence and disease-related function as a cranioencephalic unit.

## Results

### Cranial enrichment of immune cells in glioblastoma

**Fig. 1:**
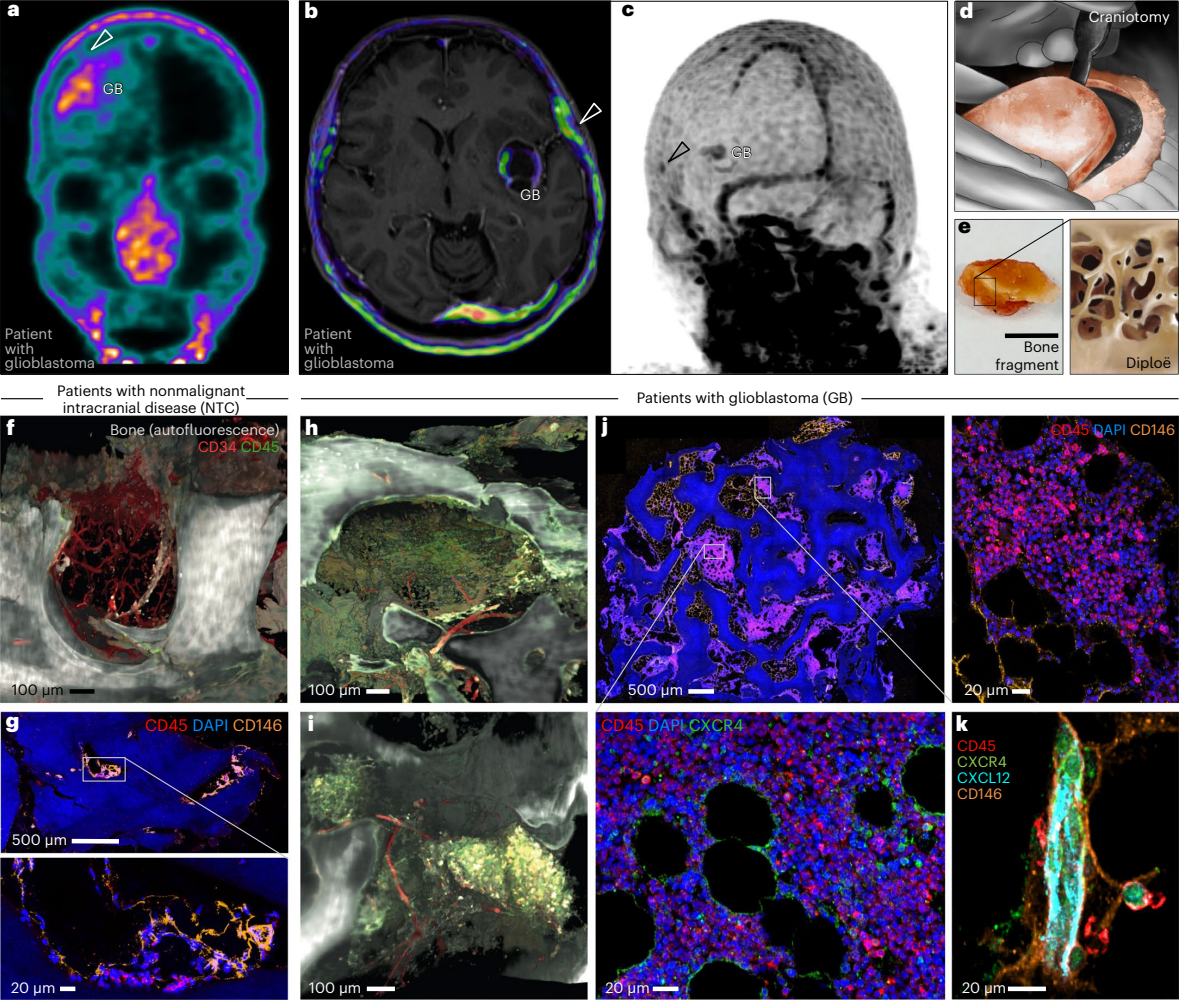
Glioblastoma-associated enrichment of immune cells in the CB. **a**, Clinical PET–CT visualizing radiolabeled CXCR4 in a coronal plane (patient 1). Arrowhead depicts focal contact between GB parenchyma and superficial cranial–meningeal compartment. Additional findings include demarcation of the nasopharyngeal mucosa and parts of Waldeyer’s pharyngeal ring. **b**, Clinical CXCR4 PET–CT in an axial plane (patient 2). Secondary fusion with MRI exposing brain anatomy and the connecting meningeal structures. Arrowhead depicts nodular enhancement ipsilateral to the GB parenchyma. Note the lower unconnected radiolabeling of the dural sinus. **c**, Three-dimensional reconstruction of PET–CT data from **b**. Arrowhead marks focal CXCR4 radiolabeling in the CB in juxtaposition to the intracerebral GB. Note the unconnected aspects of the neuro-immune interface of the dural sinus and bystander radiolabeling of the head and neck lymphatic system. **d**, Schematic representation of a craniotomy. **e**, Photograph of a representative fresh bone specimen used for the study (scale bar: 5 mm). Magnified inset: drawing of inner spongy structure. **f**, Three-dimensional rendering of light-sheet microscopy data obtained from whole-mount preparation of fixed and optically cleared clinical CB (patient c3). Note the empty aspect of fatty, aged marrow. **g**, Immunofluorescence confocal image of CB tissue section showing microvessels (CD146^+^) and limited presence of immune cells (CD45^+^) in the diploë of patient c7 (total *n* = 5 patients with nonmalignant intracranial disease; Extended Data Fig. 2). **h**,**i**, Light-sheet microscopy data as in **f**, showing accumulation of CD45^+^ immune cells surrounding CD34^+^ microvessels in the diploë of patients with glioblastoma (**h**, patient 7; **i**, patient 6). **j**, Immunofluorescence confocal image, as in **g**, demonstrating morphological appearance of tumor-associated bone cavities and accumulating immune cells (patient 12; total *n* = 4 patients; Extended Data Fig. 2). Insets detail immune and hematopoietic cell clusters (CD45^+^) and CXCR4 labeling within. **k**, Multicolor immunofluorescence confocal image capturing close proximity of CXCL12 to CXCR4^+^ cells in a CB tissue section of patient 13 (total *n* = 2 patients; Extended Data Fig. 2). Scale bars as indicated. NTC, samples from patients with nonmalignant intracranial disease; GB, samples from patients with glioblastoma. Source data

**Extended Data Fig. 1:**
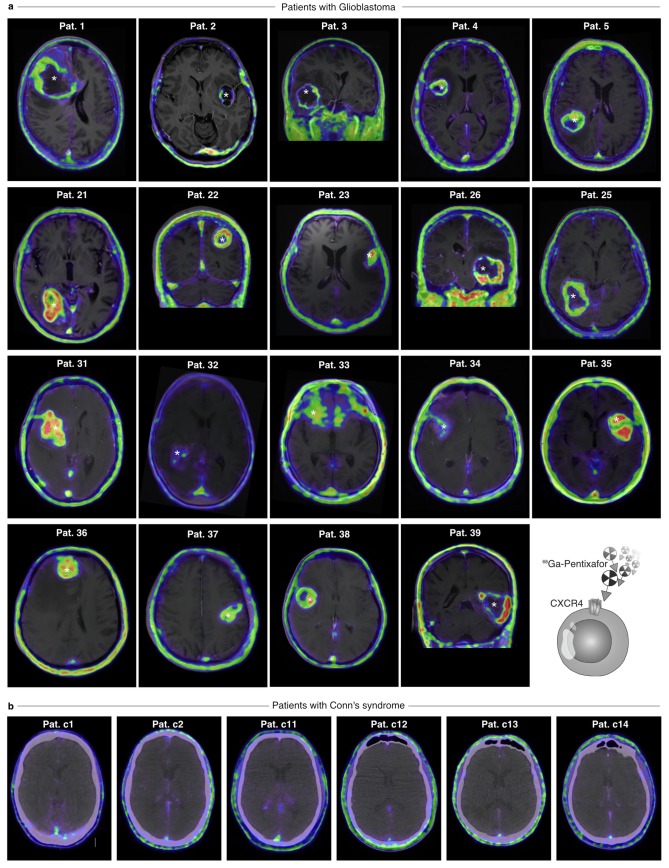
Clinical [^68^Ga]Ga-Pentixafor radiolabeling. **a**, Representative imaging results of 19 patients with newly-diagnosed glioblastoma prior to neurosurgical tumor removal (patient 1 and 2 also included in Fig. 1). Clinical radiolabeling of CXCR4 and CT/MRI fusion allows identification of glioblastoma parenchyma (asterisks) and surrounding encephalic and cranial structures. **b**, Representative Pentixafor PET-CT imaging data obtained from six patients diagnosed with Conn’s syndrome, as a control, not suffering from intracranial neoplasia. Note the absence of tracer accumulation within the cranial bone.

We used the radioligand ^68^Ga-Pentixafor as a clinical surrogate marker of immune cell presence in the CB, as it is known to act as a CXCL12 analog that binds the C-X-C motif chemokine receptor 4 (CXCR4) protein enriched in hematopoietic and immune cell niches in situ^13^. We analyzed 19 patients with glioblastoma (aged 50–83 years, median = 69 years; Supplementary Table 1) in a presurgical setting by positron emission tomography (PET). Comparing with PET data derived from six patients with Conn’s syndrome (aged 42–67 years, median = 50 years; Supplementary Table 2) as a control, we noted pronounced labeling in the CB of the patients with glioblastoma (Extended Data Fig. 1). This was contrary to our expectation that the bone marrow of the older cohort of patients with glioblastoma would be populated by aged hematopoietic cells with a reduced regenerative potential and consequently lower CXCR4 levels^12^. The pattern of Pentixafor radiolabeling frequently extended from the known accumulation within the tumor parenchyma^14^ to the adjacent tracer in the bone via ipsilateral association with bridging meningeal structures (Fig. 1a,b). No connections were observed between the various intracerebral tumor locations and the choroid plexus or the dural sinuses (Fig. 1c and Supplementary Video 1).

**Extended Data Fig. 2:**
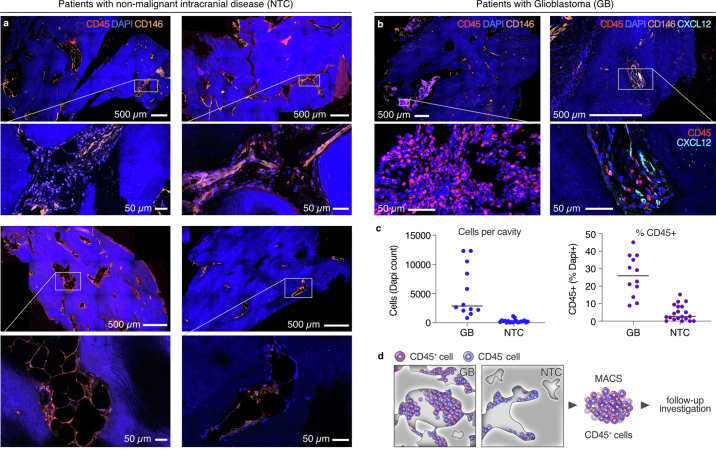
Immune cell accumulation in the cranial bone of patients with glioblastoma. **a**, Confocal immunofluorescence imaging of large CB tissue sections from four additional patients with non-malignant intracranial disease (NTC, non-tumor control; patients c15, c3, c16, c17), complementing presentation in Fig. 1f,g. Scale bars indicated. **b**, CB histological appearance of samples from two additional patients with glioblastoma (patients 29, 30), complementing presentation in Fig. 1h–k. Magnifications in the insets, scale bars indicated. **c**, Graphs present estimated immune cell frequencies in CB large tissue sections, quantified by labeling with DAPI and CD45 (data from n = 2 glioblastoma (GB) and n = 4 NTC counting cells in 12 vs. 20 cavities, respectively). **d**, Schematic illustrating derivation of vital cells from the CB cavities for follow-up investigation. Source data

Intrigued by this finding, we accessed surplus fragments of fresh bone chips derived from craniotomies under informed consent (Fig. 1d). The surgical approach of a craniotomy adjacent to the intracerebral tumor mass is indicated (1) to derive tissue for routine diagnosis and (2) for tumor resection according to the guideline-based standards of care^8^. We used whole-mount 3D light-sheet fluorescence microscopy^15^ to expose the spongy diploë within the flat bones, in which marrow characteristically resides (Fig. 1e). Samples from patients with nonmalignant intracerebral disease (*n* = 5, aged 50–83 years, median = 77 years; Supplementary Table 2) consistently displayed aging marrow^12^ (Fig. 1f,g, Supplementary Video 2 and Extended Data Fig. 2), while the diploë of patients with glioblastoma contrasted with aggregates of immune cells (*n* = 6, aged 45–80 years, median = 60 years; Supplementary Table 1). We observed extended patches of CD45^+^ cells forming solid arrangements around microvessels in the cancellous bone (Fig. 1h,i and Supplementary Videos 3 and 4). Quantitative multiplex immunofluorescence imaging on large tissue sections confirmed the observation and further revealed bone marrow-typic spatial vicinity of CXCR4 and CXCL12 in the CB (Fig. 1j,k and Extended Data Fig. 2). These data indicated a comorbid process where immune cells accumulate nearby in the proximal CB of patients with glioblastoma.

### CD8^+^ effector memory T (T_EM_) cells delineate vivid immunopathology

**Fig. 2:**
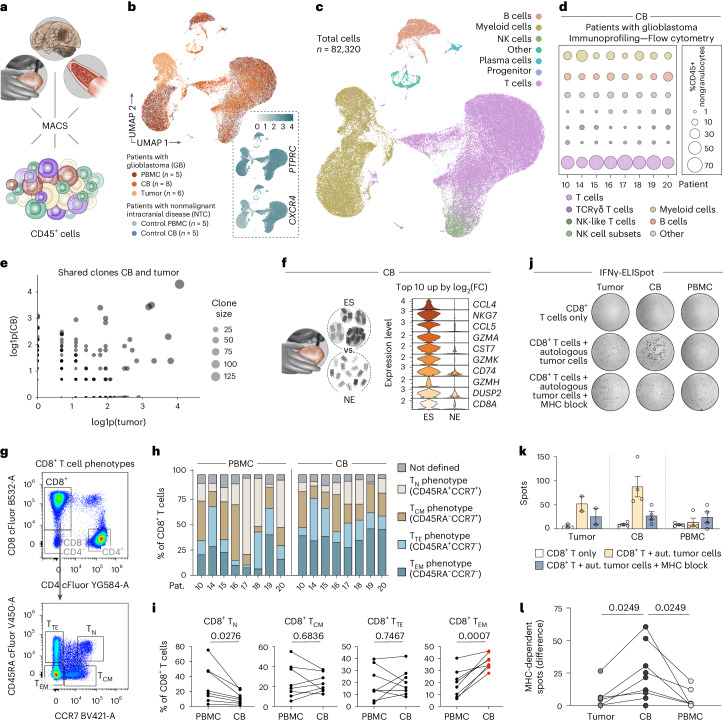
CB cellular immune profile. **a**, Schematic depicts sources of CD45^+^ immune cells. **b**, UMAP of integrated scRNA-seq data from CD45 (*PTPRC*)-expressing immune cells. Tissue sources color coded, numbers (*n*) of biosamples indicated per source. Insets visualize expression of selected genes. **c**, Overlay of SingleR- and marker-based annotation of cell types. **d**, Bubble plot summarizing prevalence of immune cell subsets among CD45^+^ nongranulocytes, by flow cytometry. **e**, Scatter plot of scVDJ data from *n* = 3 patients with glioblastoma visualizing shared T cell clonotypes between CB and tumor. Clone size visualized by number of cells per clone, each point represents a unique clone. Axes, log-transformed counts of cells (log1p). Exclusive CB or tumor clones plotted along the *y* and *x* axis, respectively. Shared clones located in the central area of the graph. **f**, Top ten differentially expressed genes (ranked by log_2_(FC)) comparing tumor-shared expanding clonotypes versus nonexpanding singlets in the CB. DEGs detected by FindMarkers() Seurat function, per default setting (two-sided Wilcoxon rank-sum test). Gene expression cutoff set to a minimum of 20% of cells. **g**, Gating strategy for profiling of CD8^+^ T cell phenotypes (T_TE_ and T_CM_). **h**, Stacked bar plot indicating phenotype distribution per patient and niche from listed patients with glioblastoma. **i**, Graphs show frequencies of phenotypes in paired samples. Two-tailed paired *t* test, *P* values indicated (*n* = 8 patients). **j**, Photomicrographs depict exemplary patterns of ELISpots using an IFNγ-based readout. **k**, Exemplary distribution of raw data from available expandable cells of one paired ELISpot analysis (*n* = 1 patient, patient 15, data points represent technical replicates per source). Data as mean ± s.e.m. **l**, Graph summarizing mean data of MHC-dependent IFNγ spots obtained from samples of patients with glioblastoma (*n* = 9). Two-tailed, paired *t* test with *P* values corrected for multiple comparisons (Benjamini–Hochberg method). UMAP, uniform manifold approximation and projection; FC, fold change; ES, expanded shared; NE, nonexpanded; T_TE_, terminal effector T cell; T_CM_, central memory T cell. Source data

**Extended Data Fig. 3:**
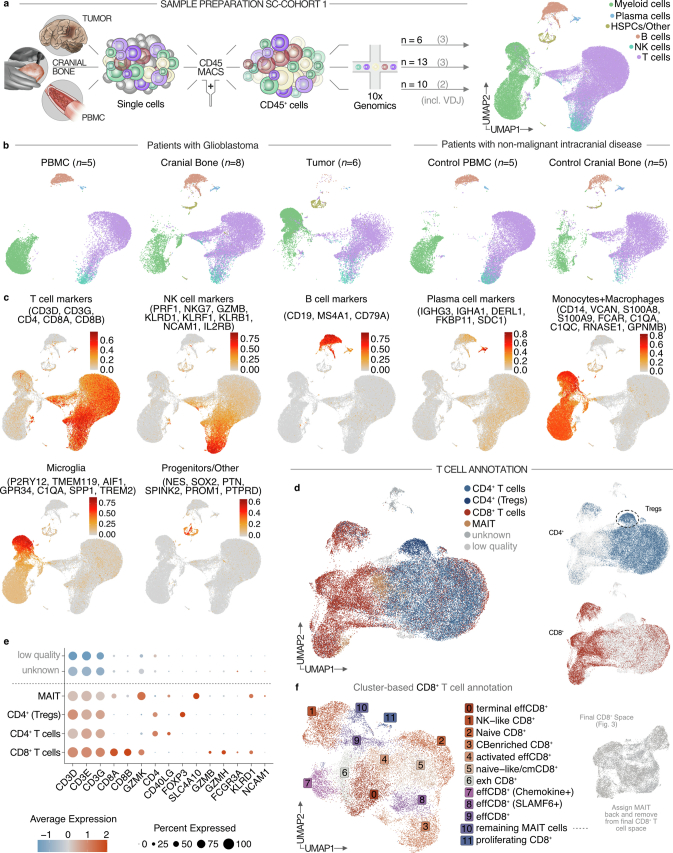
Immune cell derivation and cell type identification. **a**, Illustration of the workflow for sample processing and analysis of cells from sc-cohort 1 (see Methods). Samples from CB (n = 13), tumor tissue (n = 6) and PBMC (n = 10) enriched by CD45^+^ magnetic cell isolation. Single cells were further analyzed by scRNA-seq (10X Genomics) and integrated data were used for subsequent analyses. **b**, Split UMAP plots visualizing the distribution of annotated single cells by source. **c**, UMAP visualization of listed canonical marker genes of annotated cell types. Cells are colored by the respective gene set enrichment scores calculated via AUCell. **d**, Global UMAP of T cell types. Note, cells annotated as unknown or low quality were excluded from subsequent analyses. **e**, Bubble plot depicts the average expression levels and the fractions of cells expressing selected marker genes across the T cell types annotated in (d). **f**, Cluster-based annotation of CD8^+^ T cell subspace.

**Extended Data Fig. 4:**
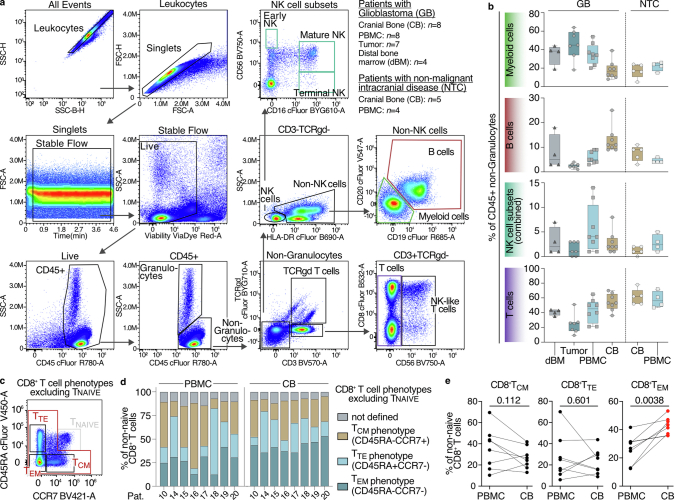
Immune cell quantification based on the Immunoprofiling Assay. **a**, Representative gating strategy. Identified phenotypes, sample origin/number as indicated. **b**, Boxplot extends from 25th to 75th percentile, displaying median and minimum/maximum ranges as whiskers, summarizing frequency data (% of CD45^+^ non-granulocytes) of indicated immune cell phenotypes, separated by source. Biological replicates, n indicated in (**a**). **c**, Representative dotplot displaying selected CD8^+^ T cell phenotypes, as indicated in red. Note, analysis excluded naive CD8^+^ T cells. **d**, Stacked bar plot indicating phenotype distribution per patient and source from listed patients. **e**, Graphs show frequencies of phenotypes in paired samples. Biological replicates (n = 8). Two-tailed paired t-test; p values indicated. Source data

**Extended Data Fig. 5:**
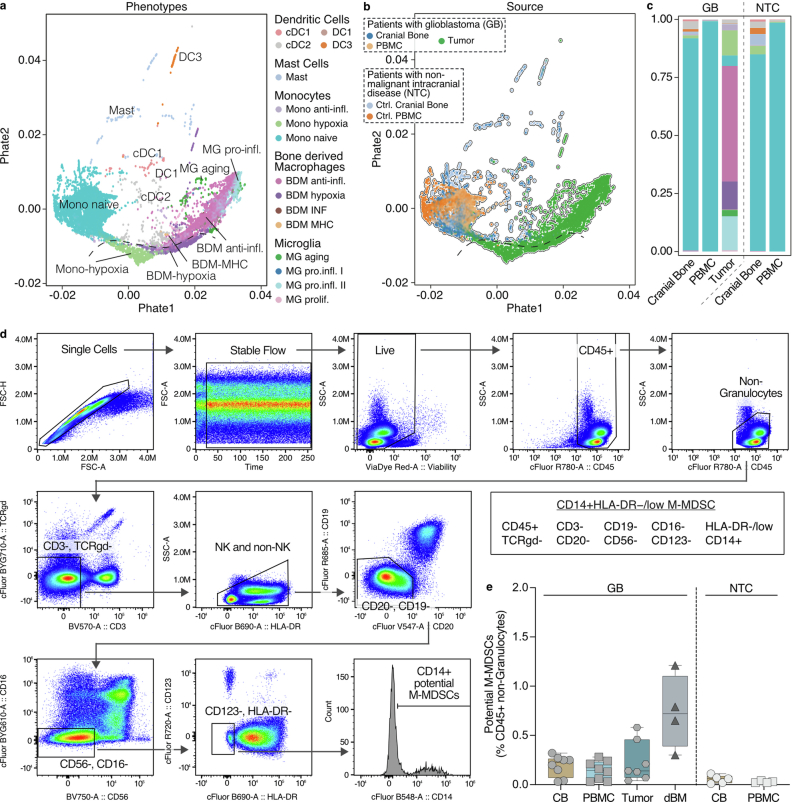
Myeloid compartment. **a**, Phate plot representing the reference-based annotation of myeloid phenotypes in the single cell data presented in main Fig. 2. **b**, Phate plot as in (**a**), displaying the distribution of myeloid cells, color coded by source. **c**, Stacked barplots indicate frequencies of myeloid cells per source and disease condition. **d**, Cytometric profiling of myeloid cells. Gating strategy used to identify potential monocytic myeloid-derived suppressor cells (M-MDSCs) utilizing the listed markers. Note, raw data derived from assay shown in Extended Data Fig. 4. **e**, Boxplot extends from 25th to 75th percentile, displaying median and minimum/maximum ranges as whiskers, summarizing frequency data of potential M-MDSCs from (d), separated by source. Biological replicate data from (n) patients: GB-CB (8), GB-PBMC (8), GB-Tumor (7), GB-dBM (4); NTC-CB (5), NTC-PBMC (4). Source data

Previous landscape analyses of the glioblastoma microenvironment have not considered immune cell niches in the CB, for example, refs. ^16–19^. Therefore, we extracted CD45^+^ immune cells by magnetically activated cell sorting from craniotomy-derived fresh surgical bone, and for comparison, from peripheral blood mononuclear cells (PBMC) and from fresh glioblastoma tissue to obtain their single-cell RNA sequencing (scRNA-seq) profiles (Fig. 2a,b). The integrated space of scRNA-seq data served as a source for marker-based cell type annotation (Fig. 2c and Extended Data Fig. 3a–c). We complemented these data by flow cytometric immunoprofiling of freshly isolated CD45^+^ cells (Fig. 2d and Extended Data Fig. 4a). Considering the importance of myeloid cells in contributing to immunosuppression in glioblastoma, and despite the preclinical evidence of skull bone marrow as a potential source of such cells^1,7^, we could not identify increased frequencies of myeloid cells in the CB, whereas these were abundant in the tumor (Extended Data Fig. 4b). scRNA-seq data suggested that CB-derived myeloid cells were mostly naive monocytes, while those in the tumor were predominantly anti-inflammatory monocytes and macrophages (Extended Data Fig. 5a–c); the latter is consistent with recent findings^20,21^. Subsequent cytometry further confirmed a low proportion of potential monocytic-myeloid-derived suppressor cells in CB samples (Extended Data Fig. 5d,e). By contrast, we surprisingly noted abundant T cell fractions among the immune cells derived from CB (Extended Data Fig. 4b).

We paired scRNA-seq and V(D)J sequencing (scVDJ) to our biosampling strategy and noticed shared clonotypes with the tumor along with an accumulation of effector-type CD8^+^ T cell transcripts within the CB (Fig. 2e,f). Quantitative phenotyping of CD8^+^ T cells confirmed consistently increased prevalence of T_EM_ phenotypes in CB (Fig. 2g–i and Extended Data Fig. 4c–e). Considering that bone marrow can be a priming site for T cell response^22^ and speculating on a distinct spatial relationship of cranial CD8^+^ T cells with the adjacent encephalic tumor mass, we asked if the proximal CB of patients with glioblastoma contained tumor-reactive cells. Hence, freshly isolated T cells were briefly expanded, magnetically sorted for CD8^+^ T cell populations and used in functional enzyme-linked immunospot (ELISpot) assays on physical contact with autologous glioblastoma cells (Fig. 2j). Relying on the secretion of interferon-gamma (IFNγ), we repeatedly observed increased major histocompatibility complex (MHC)-dependent tumor reactivity of CB CD8^+^ T cells versus paired samples from tumor or peripheral blood (Fig. 2k,l). This indicated an increased presence of tumor-reactive CD8^+^ T cells in the proximal CB, which together with abundant T_EM_ phenotypes might resemble an acute immunological response^23^.

However, the observation of lower responses from intratumoral CD8^+^ T cells challenged our perception of recirculating tumor-reactive T cells. Noting that tumor-derived T cells did not completely lack antigen specificity, we next considered the co-existence of increasingly exhausted T cell phenotypes from the tumor parenchyma in comparison to CB at the early stage of disease.

### Developmental trajectory of CD8^+^ T cell profiles

**Fig. 3:**
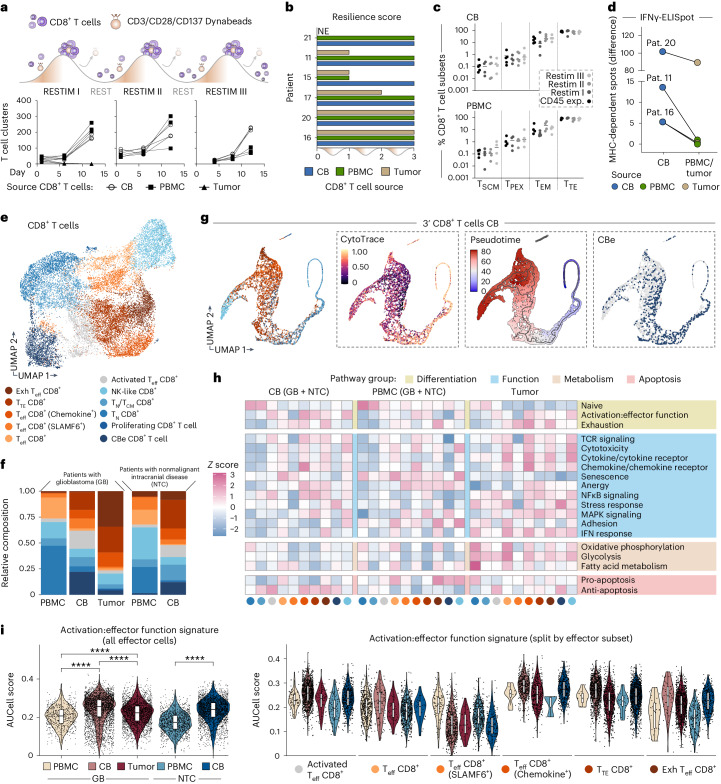
Developmental trajectory of CD8^+^ T cell response. **a**, Illustration of experimental approach used to monitor T cell aggregation. Expanded CD8^+^ T cells were restimulated (RESTIM) and allowed to rest in intermediary phases. Graphs display quantification of clusters forming at indicated time points, *n* = 3 technical replicates, patient 11. **b**, Resilience assay. Data represent successful rounds of restimulation. Experiment conducted in triplicates per patient and source. **c**, Subanalysis of **b**. Distribution of CD8^+^ T cell phenotypes by cytometry after indicated stage of stimulation (CD45 exp.) or restimulation (Restim I–III). Data points represent *n* = 4 or *n* = 5 biological replicates per condition. T cells—T_SCM_, T_PEX_, T_EM_, T_TE_. **d**, ELISpot data of specified CD8^+^ T cells after two (patient 20) or three (patients 11 and 16) rounds of restimulation in response to autologous tumor cells. Graph summarizing mean data of MHC-dependent IFNγ spots (*n* = 3 patients), paired samples indicated. **e**, UMAP of scRNA-seq CD8^+^ T cell data, color coded by annotated cell type. **f**, Stacked plot of CD8^+^ T cell data separated by condition and tissue source. CBe T cell types in dark blue. **g**, UMAP of 3′ GEX CB data from **e** generated by Palantir, based on diffusion map dimensionality reduction, color coded as in **e**. Left, continuous CytoTRACE score, from 1 (highest) to 0 (lowest) level of plasticity. Center, pseudotime calculation transitioning from blue (start) to red (end), root state manually defined. Right, CBe T cell distribution in the UMAP. **h**, Heatmap visualizing *z* scores of AUCell scores calculated using external gene signatures^27^ across CD8^+^ T cell phenotypes from **e**. **i**, Violin plot showing activation:effector function signature intensities (AUCell score) in the effector CD8^+^ T cell subtypes of **h**, split by niche and T cell subset. Boxplots display median, quartiles and values within 1.5× interquartile range as whiskers. Biological replicate data from (*n*) patients: GB-PBMC (5 patients), GB-CB (8 patients), GB-tumor (6 patients), control-PBMC (5 patients) and control-CB (5 patients). Significance calculated by two-sided Wilcoxon rank-sum test with adjusted *P* value using Holm correction (SeuratExtend); absolute values provided in Supplementary Table 3. ****P < 0.0001. T_SCM_, stem-like memory T cell; T_PEX_, progenitor exhausted T cell. Source data

**Extended Data Fig. 6:**
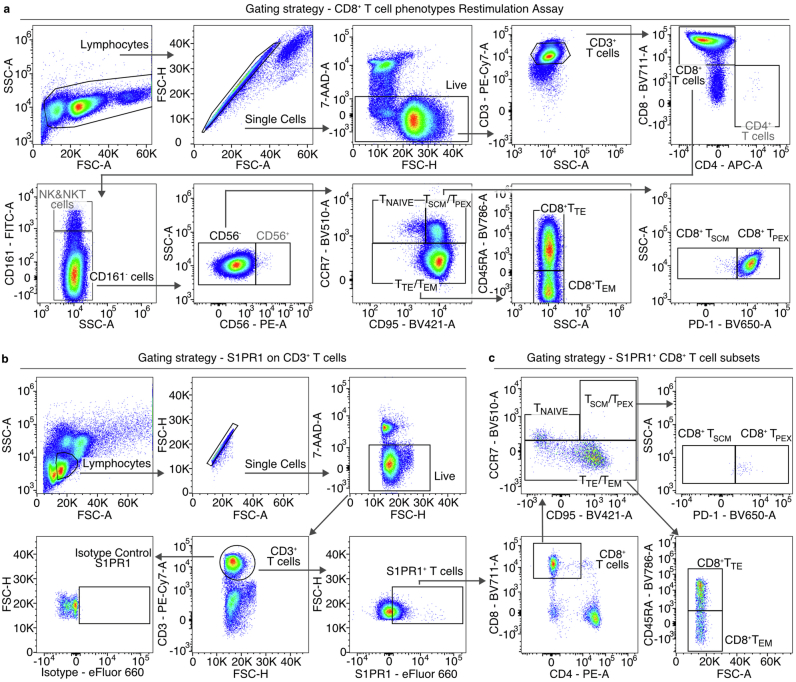
Distinct cytometric gating strategies for selected experimental approaches. **a**, Related to main Fig. 3c, re-stimulation assay. Gating used to identify CD8^+^ T cell subsets at different stages (CD45 expanded, re-stimulation I, II and III). **b**, Related to main Fig. 4b, acutely isolated CD45^+^ immune cells. Gating used to identify CD3^+^ T cells expressing S1PR1. **c**, related to main Fig. 4c. Sub-characterization of S1PR1^+^ CD8^+^ T cell phenotypes from (**b**).

We compared intra-individual levels of T cell proliferative capacity and tumor reactivity using freshly isolated and expanded CD8^+^ T cells from tumor, CB and peripheral blood of six patients with glioblastoma. In a pilot experiment, we monitored T cell aggregation as a hallmark of T cell activation observing that tumor-derived T cells were incapable of expansion on repeated stimulation while CB-derived samples maintained their proliferative potential (Fig. 3a). Standardized assessment established a resilience score for every sample indicating the continued reproductive capacity of T cell populations during three times of restimulation for 14 days, interrupted by resting periods of 7 days. Every CB-derived sample passed the test while four of six of the tumor pendants failed (Fig. 3b). We noted comparable resilience of CB- and PBMC-derived CD8^+^ T cells including a sustained ability to reproduce memory and effector T cell subsets in the experimental course (Fig. 3c and Extended Data Fig. 6a). Furthermore, we observed an enduring antitumor response of CB CD8^+^ T cells, evident by MHC-dependent tumor reactivity that continued to surpass the levels of paired tumor- and PBMC-derived populations after rounds of restimulation in the assay (Fig. 3d).

**Extended Data Fig. 7:**
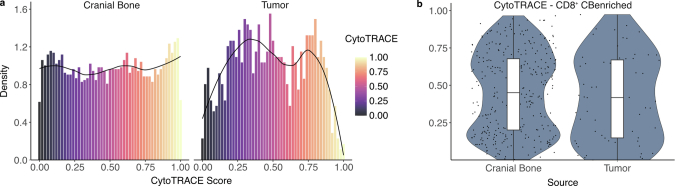
Developmental range assessment. **a**, Density plot illustrates the distribution of glioblastoma 3′ GEX CD8^+^ T cell data across the complete range of CytoTRACE scores, split by source. Note uniform distribution across all developmental stages in CB. **b**, Violin plot illustrating distribution of CBe CD8^+^ T cells across CytoTRACE scores from (**a**), split by source. Boxplots display median, quartiles and values within 1.5× interquartile range as whiskers. Biological replicate data from (n) patients: Cranial bone (5), tumor (3). No statistically significant difference detected by two-sided Wilcoxon rank sum test with p value adjustments Holm method (SeuratExtend).

To better comprehend the basis of resilience and durability of the CB T cell response, we subclustered our scRNA-seq data focusing on the transcriptomes of *n* = 18,973 CD8^+^ T cells collected from eight patients with glioblastoma and, as control, from five patients with nonmalignant intracranial disease (Fig. 3e, Extended Data Fig. 3d–f and Supplementary Tables 1 and 2). We found characteristic patterns of phenotypes by comparing the various sources of samples. In line with our hypothesis, we determined the exhausted phenotype in more than one-third of tumor-derived CD8^+^ T cells, far more prominent than in the CB or PBMC populations. We also noted distinct cellular identities enriched in the CB of patients with glioblastoma (CBenriched, CBe; Fig. 3f). Further study of the CB subspace (*n* = 6,743 cells) revealed the entire T cell developmental spectrum by Palantir. This employs diffusion map-based dimensionality reduction to better represent the differentiation trajectory of cells^24^. CytoTRACE and Pseudotime analyses, which are used to infer cellular differentiation states and dynamics of lineage specification^25,26^, confirmed the impression. The CBe CD8^+^ T cells mapped along the entire axis of Palantir-ordered phenotypes (Fig. 3g). Speculating on the presence of a distinct functional state, we benchmarked our data to recently introduced comprehensively curated T cell gene profiles^27^. This revealed anti-apoptotic and stress-related signatures, particularly enhanced in the CBe CD8^+^ T cells from CB and tumor niches (Fig. 3h). Comparable response states of tumor-infiltrating T cells were recently discussed in association with some of the most aggressive types of cancer^28^. By direct comparison, we found a broader developmental range of CD8^+^ T cell differentiation in the CB compared to tumor-derived samples, while CBe phenotypes from the two niches showed a similar distribution of developmental potential (Extended Data Fig. 7). The combined data suggested the CB as a unique niche in glioblastoma serving as a major site for differentiation of tumor-associated durable effector T cell subsets. In line with this assumption, we found an intriguing enrichment of the activation:effector function signature among the effector phenotypes in the CB (Fig. 3i), further promoting the concept of a locally driven acute CD8^+^ T cell response at this early stage of disease.

### Antitumor effect of CD8^+^ T cells from proximal CB

**Fig. 4:**
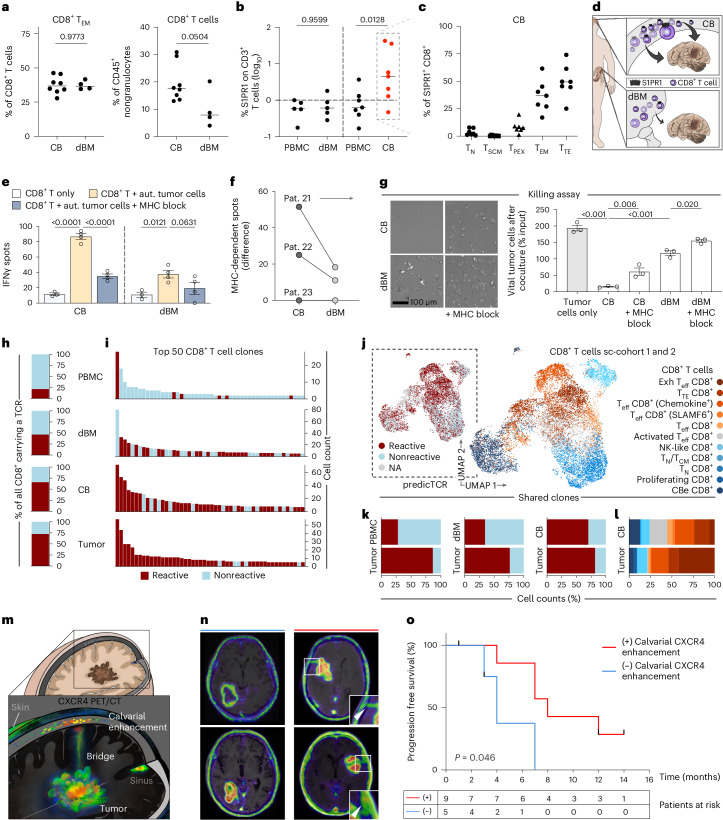
Distinctive features of CD8^+^ T cells in the proximal bone marrow. **a**–**c**, Lines indicate median, *P* values specified, (*n*) patients analyzed. **a**, Cytometry of CD8^+^ T cells from CB (8 patients) and dBM (4 patients). Two-tailed unpaired *t* test. **b**, Cytometric S1PR1 levels from freshly isolated PBMC versus dBM (5 patients); PBMC versus CB (7 patients) samples of patients with glioblastoma. One-way ANOVA corrected for multiple comparisons (Šídák test). **c**, Phenotype frequency among CB-derived S1PR1^+^ CD8^+^ cells (*n* = 7). **d**, Schematic concept. **e**, ELISpot data, split by source (patient 21). Technical replicates shown as individual dots. Mean ± s.e.m., two-way ANOVA corrected for multiple comparisons (Dunnett test), *P* values indicated. **f**, Summary graph of MHC-dependent spot mean data, as in **e**, *n* = 3 patients with glioblastoma. Paired data indicated. **g**, Killing assay. Left, phase contrast appearance at readout, after exposure to CD8^+^ T cells. Scale bar indicated. Right, graph represents percentage of viable tumor cells relative to input. Technical replicates as individual dots. Mean ± s.e.m., one of two independent experiments with similar results (patient 21). One-way ANOVA corrected for multiple comparisons (Šídák test), *P* values indicated. **h**, Frequency of predicted tumor reactivity in individual CD8^+^ T cells by predicTCR. (*n*) patients per source: PBMC (2 patients), dBM (3 patients), CB (6 patients), tumor (6 patients). **i**, Bar plots per source aligning top 50 CD8^+^ clonotypes by frequency. **j**, UMAP of CD8^+^ T cells with paired scVDJ information (*n* = 14,960), categorized by T cell subtype. Inset displays tumor reactivity by predicTCR. **k**, Frequency of predicted tumor reactivity among tumor-shared CD8^+^ clonotypes, by source. **l**, Stacked bar plots visualizing CD8^+^ T cell phenotypes among tumor-reactive clonotypes shared between CB and tumor, split per source. **m**, Illustration of sites assessed for PET–CT/MRI-specific Pentixafor labeling. **n**, Presurgical CXCR4 PET–CT data, secondary MRI fused, showing examples with (red) and without (blue) radiotracer enhancement in the CB at initial diagnosis. Insets magnify selected CB areas. Arrowheads point to radiotracer enhancement. **o**, Kaplan–Meier survival plot of patients with glioblastoma. Censored data and *P* value indicated. log-rank (Mantel–Cox) test. Source data

**Extended Data Fig. 8:**
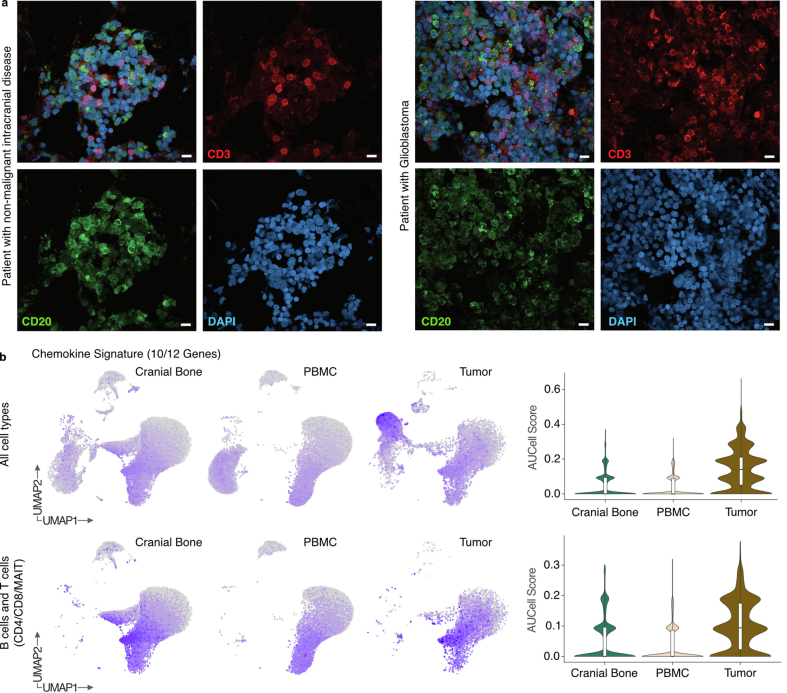
Arrangement of immune cells in the cranial bone. **a**, Immunofluorescent labeling of CD3 (red; T cells) and CD20 (green; B cells) in histological section from CB fragments of one patient with non-malignant intracranial disease (patient c6), and one patient with glioblastoma (patient 10). Nuclei were DAPI counterstained (blue). Note the lack of higher morphological organization of the tissue. Follicular arrangements, which are characteristic of matured tertiary lymphoid structures are not evident. Scale bars: 10 µm. **b**, Gene set enrichment score of a 12-chemokine reference TLS signature^29^ does not indicate enrichment in the CB single-cell data set. Scores were calculated via AUCell and depicted as UMAP, colored by score or as a violin plot, respectively, split by biological replicate data source. Cranial bone (n = 13), PBMC (n = 10) and tumor tissue (n = 6). Boxplots display median, quartiles and values within 1.5× interquartile range as whiskers. Gene set enrichment scores are shown across all annotated cell types (upper panel) or in B and T cells (CD4^+^/CD8^+^/MAIT) alone (lower panel).

To substantiate our observation of accumulating activated effector T cell types in the tumor-adjacent CB, we next considered the potential formation of tertiary lymphoid structures (TLS). TLS are known to form in association with numerous types of cancer^29^. The simultaneous presence of T cells and B cells was suggestive, but their weak structural organization in the diploë of patients with glioblastoma and the lack of characteristic single-cell gene signatures for the detection of fully developed TLS^29^ did not support the premise (Extended Data Fig. 8). On the other hand, CXCR4−CXCL12 have recently been shown to contribute to enhanced bone marrow accumulation of CD8^+^ T_EM_ and other types of memory T cells, at least during transient nutritional stress periods^30^. To assess this aspect, we collected a limited set of additional samples from the distal hip bone marrow (dBM) during neurosurgery of treatment-naive, newly diagnosed patients with glioblastoma. Comparing acutely isolated samples derived from CB and dBM we found similar relative frequencies of T_EM_ while CD8^+^ T cell fractions were increased in the CB of patients with glioblastoma, although the difference was not significant (Fig. 4a). We next studied the presence of sphingosine 1-phosphate receptor 1 (S1PR1 or S1P1) by cytometry, observing, in contrast to dBM, increased levels on T cells from the CB, particularly on the prominent CD8^+^ effector types. Among the memory-like T cells, S1PR1 was most abundant in T_EM_ (Fig. 4b,c and Extended Data Fig. 6b,c). This finding could not be anticipated from previous clinical evidence on naive T cells sequestering in the dBM at the initial stages of glioblastoma^11^. It rather suggested increased lymphoid egress^31^ from the CB (Fig. 4d), encouraging a more granular investigation of antitumor effects elicited by T cells from the cranial niche.

**Extended Data Fig. 9:**
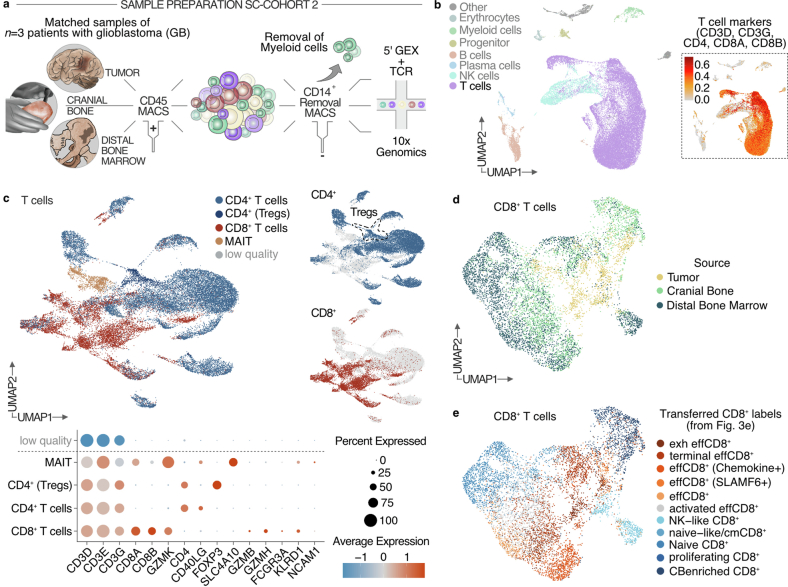
Sample preparation and annotation of sc-cohort 2. **a**, Sample processing and analysis workflow. Single cells from CB, tumor tissue and distal bone marrow (n = 3; patients 21, 22, 24) were enriched for CD45^+^/CD14^−^ cells by magnetic cell separation and further processed for scRNA-seq (10x Genomics) **b**, UMAP projection of integrated space. Inset colored according to the gene set enrichment score of canonical T cell marker genes, calculated via AUCell. **c**, UMAP of all T cells displaying annotated subtypes. Cells annotated as low quality were excluded from subsequent analyses. **d**, UMAP plot of CD8^+^ T cell subset colored by source. **e**, Annotated CD8^+^ phenotypes by label transfer from CD8^+^ T cells of sc-cohort 1 (see Fig. 3e) using singleCellNet.

**Extended Data Fig. 10:**
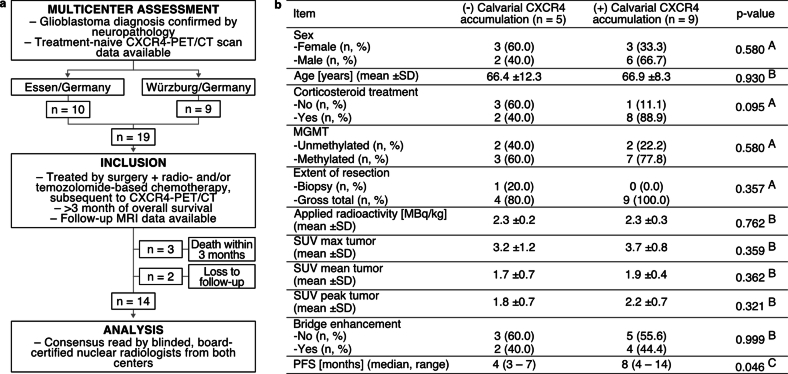
Correlation of clinical and PET-CT/MRI data. **a**, Design of study. **b**, Data considered for univariate analyses. Note that low number of cases per group (n < 10) precludes multivariate analysis. A, two-sided Fisher’s exact test; B, two-sided Student’s t-test; C, log-rank (Mantel-Cox) test. n, number of patients with glioblastoma.

Comparing tumor reactivity from bulk CD8^+^ T cell populations of CB versus dBM, we noted intra-individual differences in IFNγ release and tumor cell-killing ability (Fig. 4e–g). Using scVDJ data of acutely isolated samples (Extended Data Fig. 9), we next employed the recently introduced AI-based algorithm predicTCR^32^ to classify tumor reactivity on a single-cell level and to map their clonal distribution in the shared environments. We determined a substantial enrichment of CD8^+^ clones predicted to be tumor reactive in the CB as compared to peripheral blood and dBM that almost paralleled the frequency in the tumor niche (Fig. 4h,i). Enriching the analysis with phenotype information (Fig. 4j), we found that shared clones predicted to be tumor reactive (Fig. 4k) predominantly consist of activated and effector types in the CB versus exhausted phenotypes in the tumor (Fig. 4l). Intriguingly, we identified almost the same frequencies of tumor-reactive cells among the shared clones in CB and tumor, which might indicate that their proximity allows efficacious trafficking between the sites. Pilot evidence for the validity of this assumption was derived from the re-assessment of PET-computed tomography/magnetic resonance imaging (PET–CT/MRI)-specific radiological measures of Pentixafor in patients with glioblastoma (Fig. 4m). Among the considered parameters (Extended Data Fig. 10), only cranial and calvarial enhancement of the CXCR4 radiolabel (Fig. 4n) showed a positive correlation with improved patient outcome (Fig. 4o). Thus, the clinical imaging data that we used as an entry route into this investigation might have already contained prognostic information. Together, we interpret these findings as indicative of an immediate clinical effect mediated by an early response of tumor-reactive CD8^+^ T cells in the CB niche and their shared clonotypes in the glioblastoma tissue.

## Discussion

By combining state-of-the-art research techniques with an unconventional clinical sampling strategy, we exposed and validated tumor-associated CD8^+^ T cells in the CB of newly diagnosed, treatment-naive glioblastoma. Their effector types are characterized by an enduring tumor response, and, compared to cells from the dBM, by an increased expression of the lymphoid egress marker S1PR1. Recirculation between the proximal bone and tumor tissue is evident by the presence of shared tumor-reactive clonotypes. CXCR4 radiolabeling in the CB, in juxtaposition to the tumor, might serve as a surrogate marker indicating an association with patient survival.

Previously described low numbers of infiltrating immune effector cell types in the tissue of brain tumors have manifested the perception of a ‘cold immune phenotype’ (ref. ^10^). Current observations of clonally expanded T cells with effector properties in pediatric brain tumors^33^ and our clinical discovery of tumor-reactive CD8^+^ clonotypes in the CB, also shared with the tumor tissue of adults, challenge this concept. The accumulation of CD8^+^ T_EM_ in the proximal bone is particularly intriguing because this population of immune cells is associated with durable antitumor response in solid cancers^34,35^.

Although it has been known that the bone marrow is a major reservoir and site of recruitment for memory CD8^+^ T cells and thus could host early immune responses^22,36^, brain tumor-reactive T cells have not yet been reported in this niche. Rather, sequestration of naive T cells was described in the distal marrow^11^. This suggests that proximity plays a conceptual role in the process of early antitumor response, at least in the brain. We note as well that analysis of deep cervical tumor-draining lymph nodes in mouse models of glioblastoma showed mixed evidence of CD8^+^ tumor-reactive T cells^37–39^, suggesting that the proximal CB, in closer anatomical connection to the neuro-immune interface, may be the primary target for tumor-reactive T cell recirculation and T cell memory formation in humans.

The comprehensive exploration of CD8^+^ T cell differentiation trajectories and their cellular origins in the aged human marrow microenvironment will remain an endeavor. Further access to the niche is required to fully comprehend what presently may appear as a brain tissue-specific constellation of mechanisms of immunopathology. Advanced characterization of T cells, for example, as in refs. ^27,32,40,41^, may serve as a strategy to uncover inherent biomarkers in the dynamic course of disease and will facilitate the development of innovative diagnostic tools. Tumor-adjacent bone material as a rich source of non-exhausted tumor-reactive T cells may also have implications for the improvement of interventions, for example, related to local engaging of T cells, cellular therapies or tumor vaccination^42–46^.

Our observation was made at the time of initial diagnosis, before the onset of treatment, and may explain the survival benefit and the increase of clonal T cell diversity observed in patients with glioblastoma undergoing neoadjuvant immunotherapy compared to patients that were treated in the adjuvant setting^47,48^. Consequently, patients with an intact immunological axis, as seen in the neoadjuvant setting before craniotomy, may have a higher likelihood of responding to immunotherapy. This hypothesis is supported by the reported phenomenon, present in about one-third of newly diagnosed glioblastomas, where tumors remained stable or even decreased in volume in the waiting period between the initial diagnostic and preoperative MRI scans^49^. Therefore, the integrity of these cranioencephalic units may be pivotal for the support of immunotherapy. The preservation of these niches during treatment would require deviation from current guideline-based standards of care that enforce the transient removal of the proximal bone during neurosurgery and penetration of the site with radiotherapy^8^. Further investigation is warranted to determine the role of intact cranioencephalic units during neoadjuvant immunotherapy, requiring prospective collection of tissues from paired cohorts of patients with primary and relapsed glioblastoma. Such approaches would also yield mechanistic insight into the clinical surrogate marker CXCR4, paralleling previous preclinical work^30^, or provide a basis for the future development of alternative imaging-guided biomarkers.

Limitations of our work include the discovery-phase data restrictions on the clinical course. These data require validation through recruitment of a broad, balanced patient cohort in the prospective setting. The identity and potential clinical impact of CBe T cell states were not addressed here and need to be referred to future investigations. Interestingly, similar cell states were previously linked to immunotherapy resistance^28^. The role played by immune cell subsets other than CD8^+^ T cells warrants as well further in-depth studies along the course of disease in glioblastoma. Another interesting aspect that we did not address was the identity and the potential clinical impact of shared CD8^+^ T cell clones between tumor and dBM. Finally, our study focused solely on glioblastoma but future investigations should be expanded to other intracerebral diseases.

In conclusion, the glioblastoma-linked immune-cell niche in the human skull provides an unanticipated resource and concept of acute tumor reactivity in the proximal bone marrow. Our data advocate preservation and further exploitation of this niche and its attendant cranioencephalic units.

## Methods

### Ethics statement

Written informed consent was obtained from all participants of this study. All procedures were performed in accordance with the Declaration of Helsinki and approved by the local ethics committees (University Hospital Essen, 19-8706-BO and 22-10564-BO; University Hospital Würzburg approval, 20230824 01).

### Human biosampling

Clinical specimens were collected from patients newly diagnosed with IDH-wildtype glioblastoma, CNS WHO grade 4 (ref. ^50^), who had not undergone chemotherapy or radiotherapy. The specimens were obtained at the Department of Neurosurgery and Spine Surgery, University Hospital Essen. As control, tissue was collected from patients with nonmalignant intracranial disease (Supplementary Tables 1 and 2). At surgery, no patient suffered from acute infection or chronic inflammation. Calvarial bone chips derived during craniotomy from unplanned, intrasurgically required extensions of the burr hole or during necessary additional temporobasal decompression after craniotomy. Tumor tissue was obtained from contrast-enhanced, 5-aminolevulinic acid fluorescence, non-necrotic tumor areas by neuronavigation (Brainlab). Samples of tumor and paired bone were immediately stored in sterile Dulbecco’s Modified Eagle Medium (DMEM)/F12 (Gibco, 11320033), supplemented with antibiotics and antimycotics (2%; Gibco, 15240062). The standard collection of venous blood occurred at surgery or within 24 h. Standard dBM aspiration from posterior iliac crest was performed under general anesthesia before neurosurgery. Samples were immediately processed in the lab and registered at the Westdeutsche Biobank Essen (WBE; 22-WBE-137). Postsurgical CT scans were obtained within 24 h, and additional MRI scans of patients with glioblastoma within 72 h.

### Clinical CXCR4 radiolabeling

PET–CT imaging data (University Hospital Wuerzburg)^14^ complemented data derived from presurgical ^68^Ga-labeled CXCR4 (Pentixafor) radiolabeling of patients with glioblastoma as part of clinical care at the University Hospital Essen (Supplementary Table 1). Intravenous (i.v.) administration of Pentixafor in Würzburg and Essen used activities of 1.94 ± 0.41 MBq kg^−1^ and 2.38 ± 0.39 MBq kg^−1^, respectively, followed by imaging 72 ± 14 min and 65 ± 19 min thereafter (mean ± s.d.). Integrated data (*n* = 19 histologically confirmed glioblastoma) underwent blinded consensus read by board-certified nuclear radiologists from both centers, using equal range settings. Cranial and calvarial enhancement was defined as focal uptake in the tumor-adjacent CB and absence of uptake in the contralateral reference point. Bridging tracer enhancement was classified as clearly distinguishable tracer transition between tumor and CB exceeding brain background uptake. Tracer uptake in the skin or in the venous sinuses was not assessed. As a control, patients not suffering from brain tumors (*n* = 6; Supplementary Table 2) received i.v. Pentixafor during clinical workup in Essen with an activity of 2.13 ± 0.25 MBq kg^−1^. Imaging was performed 81 ± 10 min thereafter on a Siemens Vision PET–CT scanner and CT–MRI fusion was conducted by board-certified nuclear medicine personnel using syngo.via (Siemens Healthineers) or Brainlab’s cranial navigation software (iPlanNet).

### Tumor tissue processing

Within 30 min after resection, samples were minced and processed for derivation of primary cell cultures^51^. In parallel, single-cell suspensions were prepared^18^ by homogenizing tissue in Iscove’s Modified Dulbecco’s Medium (IMDM; Gibco, 12440053) with 0.11 DMC U ml^−1^ neutral protease (NP; Nordmark Biochemicals, S3030112) at 37 °C for ~30 min in a shaker incubator supported by intermittent resuspension. Cell suspension was filtered (35 µm cell strainer; Falcon, 352235) and washed twice with PBS (pH 7.4; Gibco, 14190169), supplemented with 0.04% BSA (Miltenyi Biotec, 130-091-376).

### Bone sample processing

Bone chips were flushed with 0.11 DMC U ml^−1^ NP in IMDM for 10–15 min (at 37 °C) followed by PBS/0.04% BSA. Filtered cell suspensions (35 µm cell strainer) were centrifuged (10 min, 300*g*) and washed once in PBS/0.04% BSA. If available, excess bone tissue was flash frozen in liquid nitrogen and stored at −80 °C.

### Blood sample and dBM processing

Blood and dBM samples arrived at room temperature in tubes containing EDTA or heparin for the isolation of PBMC or bone marrow mononuclear cells (BMMC) using Histopaque-1077 (Sigma-Aldrich, 10771) density gradient centrifugation, following the manufacturer’s protocol. Cells were washed twice in PBS/0.04% BSA.

### Selection and preservation of immune cells

Single-cell suspensions from tumor tissue, bone and blood were enriched for vital CD45^+^ cells by the REAlease CD45 (TIL) MicroBead Kit (following the manufacturer’s protocol; Miltenyi Biotec, 130-121-563). Anti-CD45 antibodies were removed and cells were either used immediately or cryopreserved at −150 °C in 50% resuspension media (40% FBS in IMDM) and 50% freezing media (30% DMSO + 40% FBS in IMDM), according to CG00039 (10x Genomics). Derived samples were labeled sc-cohort 1 (Extended Data Fig. 3). Cells of sc-cohort 2 (Extended Data Fig. 9) underwent additional magnetic myeloid cell depletion by collecting the CD14^−^ negative flow through (130-050-201).

### scRNA-seq and analysis

Cell suspensions with >85% viable cells (trypan blue exclusion) were processed for scRNA-seq using Chromium Next GEM Single Cell 3′ Reagent Kit v3.1 and 5′ Reagent Kit v2 (10x Genomics, CG0000315 and CG0000331). Subsequent to quality control (2100 Bioanalyzer, Agilent), paired-end sequencing of pooled libraries was conducted on a NovaSeq 6000 System (Illumina). Reads were aligned to the hg38 human reference genome (2020) using Cell Ranger (v.7.0.1). The 5′ data, integrating V(D)J repertoire and gene expression, were processed with cellranger multi pipeline using 10x Genomics hg38 and V(D)J reference (7.0.0, GRCh38).

Analyses were performed in R (v4.2.0) on raw 3′ and filtered 5′ multi-output data. Using Seurat package (v.4.3.0)^52^, normalized cells (SCTransform) were filtered to remove cells with <500 or >7500 nFeature_RNA counts, or >15% mitochondrial genes and to identify doublets (DoubletFinder, v.2.0.3)^53^. The 3′ GEX (*n* = 21) and 5′ GEX/scVDJ (*n* = 8) Seurat objects (sc-cohort 1; Extended Data Fig. 3) were merged, cleaned of doublets and normalized regressing out mitochondrial percentage per cell and cell-cycle scores. Data integration used Harmony (v.0.1.1)^54^ by patient, followed by Seurat FindNeighbors (dims = 1:15) and FindClusters function (resolution = 0.6) with data visualization via RunUMAP (dims = 1:15; Fig. 2b,c). Cell-type annotation of integrated data was performed using SingleR (v.1.10.0)^55^ and marker-based identification via Seurat’s FindAllMarkers function and subsequent literature search. Expression of canonical marker gene sets was confirmed and visualized by gene set enrichment scores (AUCell score, v.1.18.1)^56^ (Extended Data Fig. 3c).

### Myeloid cell compartment

Myeloid cell subset was refined by removing falsely clustered T cells (*CD3D* < 0.1), followed by normalization and data integration. Cell type annotation used Azimuth tool^52^ (v1.0.2) with GBMap dataset^57^. Only myeloid cells at annotation level 3 were kept, excluding cells expressing *CD3*, *GFAP*, *OLIG1*/*OLIG2* or *RBFOX3*. The refined dataset was integrated by Harmony, followed by dimensional reduction using PHATE^58^ (v1.0.7) and cell-type identification via shared-nearest neighbor clustering.

### Tumor-shared clonotypes in the CB

For integrating scVDJ information, TCRA/TCRB nucleotide sequences were assigned to T cells using Cell Ranger’s filtered contig annotation data (patients 4, 15 and 16; sc-cohort 1) and combineExpression function of scRepertoire (v.1.11.0)^59^. Differentially expressed genes (DEGs) were detected from tumor-shared clones ( ≥ 2 cells) versus nonexpanding singlets in the CB niche using Seurat’s FindMarkers() function with min.pct=0.2 (20% of cells). The top 10 DEGs (ranked by log_2_(FC)) were visualized using Seurat’s VlnPlot.

### CD8^+/−^ T cell compartment (sc-cohort 1)

T cells were subset removing falsely clustered myeloid cells (*CD68* < 0.01), normalized and data integration reperformed. Using Seurat’s FindNeighbors (dims = 1:15), FindClusters (resolution = 0.5) and RunUMAP (dims = 1:15) functions, CD8^+^/CD4^+^ T cells, CD4^+^ T_reg_ cells and MAIT cells were distinguished by cluster-based marker gene expression (Extended Data Fig. 3d,e). Cells lacking T cell genes (‘unknown’) or displaying high mitochondrial gene expression (‘low quality’) were excluded from analyses. *CD8*^*+*^ and *CD4*^*+*^ clusters were subsetted and remaining *CD4-* or *CD8*-expressing cells were removed (*CD4* < 0.01/*CD8A* and *CD8B* < 0.01). A total of 6,550 cells remained unassigned. Their identity was determined by cluster-independent *CD4* or *CD8A*/*CD8B* expression. Identified (*CD4* < 1 × 10^−15^ and *CD8A*/*CD8B* > 1 × 10^−15^) CD8^+^ cells (*n* = 4,876) were added to the CD8^+^ T cell subspace for further analyses. Cells were normalized and integrated, followed by Seurat’s FindNeighbors (dims = 1:10), FindClusters (resolution = 0.5) and RunUMAP (dims = 1:15) commands for data visualization. Highly variable genes of the 11 distinct clusters were extracted via FindAllMarkers, and cellular identities were manually annotated (Extended Data Fig. 3f). Remaining MAIT cells, not belonging to CD8^+^ T cell subset, were re-assigned to the global T cell space (Extended Data Fig. 3d–f). Normalization and data integration revealed the final CD8^+^ T cell space (*n* = 18,973).

### CB subspace assessment

The 3′ GEX CD8^+^ T cell subset of CB (*n* = 6,743 cells) was normalized (NormalizeData) using Seurat (v4.1.1), followed by FindVariableFeatures, ScaleData (default parameters) and RunPCA functions (npcs = 100). Following data integration by patient (Harmony), cells were ordered by differentiation trajectory using Python package Palantir^24^ (v1.0.1), visualized by RunUMAP (dims = 1:4). A numeric vector, predicting cellular status from least (1.0) to most (0.0) differentiated was generated from the RNA matrix by CytoTRACE^25^ (v.0.3.3). Pseudotime analysis was conducted using Monocle 3 (ref. ^60^; v.1.3.1). Following conversion into a CDS object using as.cell_data_set from SeuratWrappers, the cluster_cell and learn_graph functions from Monocle were applied. Location of naive CD8^+^ T cells was used to specify root node (order_cells function). Combined 3′ GEX CD8^+^ T data were used to compute cellular CytoTRACE scores of tumor and CB, visualized with ggplot2 (v.3.4.3; Extended Data Fig. 7a). VlnPlot2 (SeuratExtend v.0.6.0)^61^ was used to plot and compare (Wilcoxon test) CytoTRACE scores of CBe CD8^+^ T cells from both sources (Extended Data Fig. 7b).

### Benchmarking to external signatures

Gene set enrichment scores of 19 curated CD8^+^ T cell gene signatures^27^ were computed by AUCell. *Z* scores across phenotypes and sources were calculated via CalcStats (SeuratExtend) and visualized as heatmap (Fig. 3h). Effector phenotypes were isolated and their activation:effector function signature^27^ AUCell score visualized (VlnPlot2, Seurat Extend).

### Subanalysis of sc-cohort 2

Preprocessing of scData included removal of cells with <500 or >7500 nFeature_RNA counts, >15% mitochondrial genes and doublets before data integration. Normalization (SCTransform), including regression of mitochondrial read and cell cycle scores, and Harmony by patient was executed before FindNeighbors (dims = 1:15), FindClusters (resolution = 0.4) and RunUMAP (dims = 1:15) functions for data visualization (Extended Data Fig. 9b). SingleR and AUCell score of canonical T cell genes were employed to identify CD8^+^/CD4^+^ T cells, CD4^+^ T_reg_ cells and MAIT cells (Extended Data Fig. 9c). Assignment of scVDJ information used Cell Ranger’s filtered contig annotation data and combineExpression function (scRepertoire). CD8^+^ T cells of sc-cohort 2 were subset and annotated by label transfer of sc-cohort 1 using singleCellNet^62^ (v.0.1.0; Extended Data Fig. 9d,e).

### Tumor reactivity prediction

Gene count matrix was imported into R v4.1 and normalized using SCTransform on all genes (Seurat v.4). Normalized data was imported in Python with reactivity predicted by predicTCR^32^ model under xgboost (v1.7.4). Probability of reactivity was averaged for each clonotype, and threshold was determined using Fisher–Jenks natural break optimization. Clones with reactivity scores above threshold were designated as reactive and vice versa. For visualization, scVDJ data from sc-cohort 1 (patients 4, 15 and 16) and sc-cohort 2 (patients 21, 22 and 24) were integrated (*n* = 14,960). FindNeighbors (dims = 1:10), FindClusters (resolution = 0.5) and RunUMAP (dims = 1:10) functions were executed and data were visualized as UMAP.

### T cell expansion

T cells were expanded from CD45^+^-enriched cells in T cell activation media (RPMI 1640 (Gibco, 72400021), human AB serum (10%; Sigma-Aldrich, H5667), sodium pyruvate (1 mM; Gibco, 11360039), β-mercaptoethanol (50 µM; Gibco, 21985023), antibiotic–antimycotic (1%), recombinant IL-2 (1000 U ml^−1^, 200-02), IL-15 (10 ng ml^−1^, 200-15) and IL-21 (10 ng ml^−1^, 200-21, all Peprotech)), similar to ref. ^63^. T cells expanded for 14–21 days in 96-well plates (Corning, 3596) with human T-activator CD3/CD28/CD137 Dynabeads (Gibco, 11163D) in a 1:5–10 bead-to-cell ratio. Before analyses, CD8^+^ T cells were enriched by magnetic separation (Miltenyi Biotec, 130-096-495), immediately used or stored at −150 °C.

### ELISpot assays

Cellular IFNγ release (R&D Systems, EL285 and SEL285) was detected by incubating 10,000–20,000 bulk CD8^+^ T cells and 5,000–10,000 autologous tumor cells in 96-well plates (2:1, effector-to-target ratio). Autologous, short-term expanded tumor cells (passage 4–7) were prestimulated with IFNγ (1 µg ml^−1^; Peprotech, 300-02) for 48 h. T cells rested in reduced cytokine concentrations (20 U ml^−1^ IL-2, 1 ng ml^−1^ IL-15, 1 ng ml^−1^ IL-21) for at least 3 days and overnight in cytokine-free media. ELISpot assays were performed according to the manufacturer’s instructions after 24–48 h of co-incubation. MHCI/MHCII blockade was achieved by pre-incubating tumor cells with 5 µg ml^−1^ anti-HLA-DR (clone L243) and 5 µg ml^−1^ anti-HLA-A, anti-HLA-B and anti-HLA-C (clone W6/32) antibodies (BioLegend, 307648 and 311428) for 1 h. Background controls included wells with only CD8^+^ T cells or tumor cells. Spots were counted using ELISpot reader (AID iSpot, AID Autoimmun Diagnostika) and analyzed with Fiji Software (v1.0). MHC-dependent spots are defined as

$$\begin{array}{ll}{\rm{number}}\,{\rm{of}}\,{\rm{spots}}({\rm{CD}}8^{+}{\rm{T}}\; {\rm{cells}}+{\rm{autologous}}\; {\rm{tumor}}\; {\rm{cells}})\\-{\rm{number}}\,{\rm{of}}\,{\rm{spots}}({\rm{CD}}8^{+}{\rm{T}}\; {\rm{cells}}+{\rm{autologous}}\; {\rm{tumor}}\; {\rm{cells}}+{\rm{MHC}}\; {\rm{block}})\end{array}$$

### Restimulation experiments

T cell activation was monitored by T cell clustering/aggregation during restimulation^64^. Expanded T cells rested in reduced cytokine conditions (see above) for at least 72 h. Restimulation cycles involved seeding 10,000 T cells in 96-well plates in activation media and CD3/CD28/CD137 Dynabeads (1:2, bead-to-cell ratio). Resilience assay evaluated CD8^+^ T cell fitness by counting successful restimulation cycles. Three 14-day-restimulation cycles, followed by 7 days of rest were performed in triplicates per patient and source. A restimulation cycle was successful if the mean cell count across all three wells exceeded the input of 10,000 cells per well. Resting T cells were cryopreserved in 80% FBS and 20% DMSO after expansion and restimulation.

### Killing assay

Adapting protocols from refs. ^65,66^, we enriched tumor-reactive T cells by incubating 20,000 resting CD8^+^ T cells with 5,000 IFNγ stimulated autologous tumor cells on anti-CD28-coated 96-well plates (4 µg ml^−1^; BioLegend, 302934). Media consisted of one-fourth of maintenance media for primary tumor cells^51^ + three-fourths of T cell activation media. Cocultures were fed every other day and T cell outgrowth was incubated on fresh tumor cells weekly (up to 4 weeks). Derived T cells were used for the killing assay. Briefly, for patient 21, 20,000 tumor-reactive T cells were incubated at week 4 with 5,000 autologous tumor cells labeled with 1 µM CellTracker Red (Invitrogen, C34552) supplemented with caspase 3 substrate (NucView Biotium, 10402), according to manufacturer’s protocol. After 7 days, T cells were gently removed and live adherent tumor cells were detected (Nyone, Synentec) using 1 µg ml^−1^ Hoechst 33342 (Thermo Fisher Scientific, 62249). Celltracker signals and cellular morphology discriminated tumor cells and T cells, excluding Caspase-positive cells. MHCI blockade was achieved by pre-incubating tumor cells with 10 µg ml^−1^ anti-HLA-A, anti-HLA-B and anti-HLA-C (W6/32) antibodies for 1 h. During the assay, cells were fed once with fresh media and blocking antibodies at day 4.

### Flow cytometry

Spectral flow cytometry-based immunoprofiles were detected using Cytek 25-Color Immunoprofiling Assay (Cytek Biosciences, R7-40002), with 18 cFluor reagents supplemented with seven antibodies from BioLegend (900004160) and ViaDye Red Fixable Viability Dye (Cytek), according to manufacturer’s protocol. Cryo-conserved single cells were thawed and washed twice before antibody labeling. Viability dye was used at 250 nM before blocking (Human TruStain FcX, BioLegend) and subsequent antibody labeling. Samples were measured on a Cytek Aurora flow cytometer in 5 l setup (16UV-16V-14B-10YG-8R), acquiring spectral profiles by SpectroFlo software (v3.0.3, Cytek). Unmixing was performed using the manufacturer’s recommended reference controls, with autofluorescence extraction enabled. Cell populations were quantified by recommended enhanced gating strategy (Cytek). Alternative gating to identify potential M-MDSCs was carried out via FlowJo (v10.9.0).

For phenotyping, indicated CD8^+^ cells from resilience assay were thawed, washed and incubated for 5 min with Fcγ receptor binding inhibitor (BD Pharmingen, 564220) before antibody labeling. The antibody cocktail consisted of BV421-CD95 (305623), BV711-CD8 (344733), BV510-CCR7 (353231), APC-CD4 (317415), FITC-CD161 (339905), PE-Cy7-CD3 (344815) and BV650-PD-1 (329949; all BioLegend), as well as BV786-CD45RA (563870) and PE-CD56 (555516) from BD Biosciences (all diluted at 1:20). Viability verified using 7AAD (Invitrogen, 00-6993-50). Cytometric profiling of S1PR1 on T cells was performed accordingly, by Fc-block and antibody panel (all diluted at 1:20)— BV421-CD95 (305623), BV711-CD8 (344733), BV510-CCR7 (353231), PE-CD4 (317410), PE-Cy7-CD3 (344815) and BV650-PD-1 (329949; all BioLegend), as well as BV786-CD45RA (BD Biosciences, 563870) and eFluor660-S1PR1 (50-3639-42) or respective Isotype Control (50-4714-82; both Thermo Fisher Scientific). Samples were incubated on ice in the dark for 30 min, washed and measured using FACS Celesta and FACS Diva software (v8.0.1.1, BD Biosciences), with FlowJo sub-analysis (v10.9.0). Gating strategies are depicted in the corresponding Extended Data Figs. 4–6.

### Whole-mount staining and optical clearing

CB samples were fixed in 4% PFA (in PBS, pH 7.4) overnight at 4–8 °C and blocked (5% DMSO, 0.1% Tween 20, 1% BSA and 5 mM EDTA in PBS) for two days at room temperature. Immunofluorescence labeling was performed with PE/Dazzle594-CD45 (304052) and AlexaFluor647-CD34 (343508) BioLegend antibodies diluted 1:200 in blocking buffer for 5 days at room temperature. Samples were washed twice with 5% DMSO and 0.1% Tween 20 in PBS for 1 day at room temperature, respectively. Optical tissue clearing was performed by established methods^15^. Briefly, dehydration in increasing ethanol concentrations of 50%, 70% and 100% (room temperature, 1 day each) was followed by optical clearing in ethyl cinnamate (ECi; Sigma-Aldrich, 112372) at room temperature to achieve complete transparency.

### Light-sheet fluorescence microscopy

ECi-cleared CB was imaged via light-sheet fluorescence microscopy, using a LaVision BioTec Ultramicroscope Blaze (Miltenyi Biotec and LaVision BioTec) with supercontinuum white light laser (460–800 nm), seven excitation and emission filters covering 450–865 nm, AndorNeo sCMOS Camera with pixel size of 6.5 × 6.5 µm^2^ , and ×1.1 (NA 0.1), ×4 (NA 0.35) and ×12 (NA 0.53) objectives with magnification changer ranging from ×0.66 to ×30. Cleared samples were immersed in ECi in a quartz cuvette and imaged using excitation (ex) and detection band-pass emission (em) filter settings—tissue autofluorescence, ex 500/20 nm, em 535/30 nm; CD45-PE-Dazzle594, ex 560/40 nm, em 650/50 nm; CD34-AlexaFluor647, ex 630/60 nm, em 680/30 nm. The z-step size was set to 5 or 10 µm based on the selected light-sheet NA. Depending on the objectives, optical zoom factor varied from ×4 to ×12, with a digital zoom factor of ×1. Data were processed with visualization tools from Imaris (Bitplane, v9.7.1).

### Confocal laser scanning microscopy to assess TLS formation

CB samples were decalcified in 14% EDTA-free acid solution (pH 7.2) for 14 days at room temperature, washed with PBS, embedded in O.C.T. Compound (Sakura, 4583) and snap frozen. Tissue sections of 20 µm were generated on a CryoStar NX70 (Thermo Fisher Scientific) using Kawamoto’s film method (Section Lab, Cryofilm type 2C9) and stored at −20 °C. For immunofluorescence labeling, tissue sections were blocked (1% BSA, 0.1% Tween 20 and 0.1% DMSO in PBS) for 1 h at room temperature, and incubated with PE/Dazzle594 CD3 (1:100; BioLegend, 300450), AlexaFluor488 CD20 (1:100; Thermo Fisher Scientific, 53-0202-80) and DAPI (1:500; Carl Roth, 6335.1) in blocking buffer overnight (4–8 °C). Samples were washed thrice with washing buffer for 15 min at room temperature, and once with distilled water, covered with mounting medium (Agilent Dako, S3023) and imaged via high-resolution confocal laser scanning microscopy on a Leica TCS SP8 confocal laser scanning microscope equipped with acousto-optic tunable filters, an acousto-optical beam splitter, internal hybrid detectors (HyD SP) with use of an LMT200 high precision scanning stage. A Leica HC PL APO ×63/1.20W CORR objective combined with a digital zoom factor of 1.0 was used for imaging of sequential scans as follows: (1) CD20-AlexaFluor488, ex 488 nm (argon laser), em 500–550 nm; (2) CD3-PE/Dazzle594, ex 561 nm, em 600–650 nm and (3) Dapi, ex 405 nm, em 450–500 nm, with the last two being excited by a diode-pumped solid-state laser. The 3D reconstruction used Imaris software (v9.7.1, Bitplane) at maximum intensity projection.

### Quantitative multiplex immunofluorescence imaging

CB samples were fixed with 4% methanol-free formaldehyde (Thermo Fisher Scientific) overnight with rotation at 4 °C. Decalcification (10% EDTA, pH 8; Sigma-Aldrich) for 14 days at room temperature with stirring was followed by dehydration (overnight) and paraffin embedding. Tissue sections of 10 µm were cut (pfm Slide 4004 M sledge microtome), deparaffinized, rehydrated and antigen-retrieved according to the manufacturer’s instructions (Agilent Technologies). Sections were blocked and permeabilized with TBS (0.1 M Tris, 0.15 M NaCl, pH 7.5) containing 0.05% Tween 20, 20% DMSO (Sigma-Aldrich) and 10% donkey serum (Jackson ImmunoResearch) for 15 min at room temperature. Antibodies (1:25) and DAPI were diluted in DAKO EnVision FLEX diluent (Agilent Technologies). Primary antibodies (CD45—Bio-Rad, MCA345G; CD146—R&D, AF932; CXCR4—Thermo Fisher Scientific, PA3-305) were applied overnight. Secondary antibodies (donkey anti-rabbit 488, anti-goat 555, anti-rat 594 (all Biotium)) were incubated for 5 h and DAPI (Thermo Fisher Scientific) was applied before mounting (Vector Laboratories, H-1400-10). Labeled sections were imaged on a Leica Stellaris 8 laser scanning confocal microscope equipped with 2x HyD-S, 2x HyD-X and one HyD-R detectors and two laser lines (405 and white-light laser) using ×20 multiple-immersion objective (NA 0.75, FWD 0.680 mm) at 400 Hz, 8-bit with 1024 × 1024 resolution.

### Statistics and reproducibility

Statistical methods, sample size and replication for each experiment are indicated in the figure legends. Flow cytometry and ELISpot statistical analyses were performed using GraphPad Prism v9.5.1 or Microsoft Excel v16.79.2. Statistical analysis of survival data was executed in SPSS (v.29.0.2.0). For collection of tissue samples and clinical imaging data, no statistical method was used to predetermine sample size, but our sample sizes are similar to those reported in previous publications^67–69^. Tissue samples were collected consecutively. The sex of a patient was self-reported. No gender information was collected and sex was not considered in the study design. scRNA-seq data with low quality (see above) and patients not meeting inclusion criteria for survival analysis (Extended Data Fig. 10a) were excluded from the study. Experiments were not randomized. Except for PET data association with patient survival (Fig. 4m–o and Extended Data Fig. 10), data collection and analysis were not performed blind to the conditions of the experiments. In parametric statistical tests, data distribution was assumed to be normal but this was not formally tested.

### Reporting summary

Further information on research design is available in the Nature Portfolio Reporting Summary linked to this article.

## Online content

Any methods, additional references, Nature Portfolio reporting summaries, source data, extended data, supplementary information, acknowledgements, peer review information; details of author contributions and competing interests; and statements of data and code availability are available at 10.1038/s41591-024-03152-x.

## Supplementary information

Supplementary Information Supplementary Tables 1–3.

Reporting Summary

Supplementary Video 1 Three-dimensional view of maximum intensity projection of CXCR4 radiolabeling in a patient with glioblastoma (patient 2; Fig. 1c).

Supplementary Video 2 Three-dimensional view of a whole-mount immunofluorescence preparation of a CB fragment of a patient with nonmalignant intracerebral disease (patient c3; Fig. 1f).

Supplementary Video 3 Three-dimensional view of a whole-mount immunofluorescence preparation of a CB fragment of a patient with glioblastoma (patient 7; Fig. 1h).

Supplementary Video 4 Three-dimensional view of a whole-mount immunofluorescence preparation of a CB fragment of a patient with glioblastoma (patient 6; Fig. 1i).

## Source data

Source Data Figs. 1–4 and Extended Data Figs. 2, 4 and 5 Source data Figs. 1f–k, 2d,f,h–l, 3a–e and 4a–c,e–g,j,o and Extended Data Figs. 2a–c, 4b,d,e and 5a,e.

## Data Availability

scRNA-seq data were deposited into NCBI Gene Expression Omnibus (GEO) and are available under accession GSE233304. The hg38 human reference genome and the human V(D)J reference (GRCh38) are available at https://www.10xgenomics.com/support/software/cell-ranger/latest/release-notes/cr-reference-release-notes#cr7-0. All other data and materials are available in the manuscript and supplementary data. Source data are provided with this paper.

## Code availability

There was no custom computational code or software developed for this study. Analyses were performed with publicly available software packages as described in the Methods section.
