# Supplementary material for: Cranioencephalic functional lymphoid units in glioblastoma
Source: Nat Med. 2024 Jul 31;30(10):2947–56. doi: 10.1038/s41591-024-03152-x (PMC11485206; doi:10.1038/s41591-024-03152-x)
Supplement: Supplementary file 1 — Supplementary Tables 1–3. [file 41591_2024_3152_MOESM1_ESM.pdf]

nature medicine

Supplementary information https://doi.org/10.1038/s41591-024-03152-x

Cranioencephalic functional lymphoid units in glioblastoma

In the format provided by the authors and unedited

Supplementary Information File

Dobersalske et al., Cranioencephalic functional lymphoid units in glioblastoma.

Captions for Supplementary Tables 1 - 3

Supplementary Table 1. Data and biosample use of patients with glioblastoma.

Supplementary Table 2. Data and biosample use of patients with non-malignant intracranial disease or Conn's Syndrome.

Supplementary Table 3. Wilcoxon test data, related to Fig. 3i.

Supplementary Table 1: Data and biosample use of patients with glioblastoma

Identifier	CXCR4 PET/CT	Histology (IF)	Lightsheet	scRNA-Seq (CD45+) sc-cohort 1			scRNA-Seq (CD45+ CD14-) sc-cohort 2			Functional T cell assay	Immuno-profiling	Restimulation	Functional assay Restimulation	S1PR1	Medical center	Diagnosis	MGMT	ATRX	Age	Sex
				Cranial Bone	PBMCs	Tumor	Cranial Bone	Tumor	distal BM											
Patient 1	x			x	x	x									University Hospital Essen	Glioblastoma, IDH-WT (CNS WHO grade 4)	Met	Wt	50	m
Patient 2	x			x	x	x											Met	Wt	68	f
Patient 3	x																Met	Wt	64	m
Patient 4	x			x+VDJ		x+VDJ											Met	Wt	62	m
Patient 5	x																Met	Wt	72	m
Patient 6			x														Met	Wt	45	f
Patient 7			x	x													Met	Wt	80	f
Patient 8				x													Unmet	Wt	54	m
Patient 9				x	x	x											Met	Wt	65	m
Patient 10		x								x	x						Unmet	Wt	65	f
Patient 11										x		x	x				Unmet	Wt	58	m
Patient 12		x															Met	Wt	47	f
Patient 13		x															Unmet	Wt	64	f
Patient 14											x						Met	Wt	77	f
Patient 15				x+VDJ	x+VDJ	x+VDJ				x	x	x		x			Unmet	Wt	70	f
Patient 16				x+VDJ	x+VDJ	x+VDJ				x	x	x	x	x			Unmet	Wt	53	m
Patient 17										x	x	x		x			Unmet	Wt	54	f
Patient 18											x						Unmet	Wt	75	f
Patient 19											x			x			Met	Wt	75	f
Patient 20										x	x	x	x				Unmet	Wt	60	f
Patient 21	x						x+VDJ	x+VDJ	x+VDJ	x	x	x		x			Unmet	Wt	83	f
Patient 22	x						x+VDJ	x+VDJ	x+VDJ	x	x			x			Met	Wt	70	m
Patient 23	x									x				x			Met	Wt	79	m
Patient 24							x+VDJ	x+VDJ	x+VDJ		x			x			Met	Wt	66	m
Patient 25	x										x			x			Met	Wt	56	f
Patient 26	x													x			Unmet	Wt	66	f
Patient 27														x			Met	Wt	52	m
Patient 28														x			Met	Wt	68	f
Patient 29		x															Met	Wt	70	m
Patient 30		x															Unmet	Wt	55	m
Patient 31	x														University Hospital Wuerzburg	Glioblastoma (WHO IV), PMID: 26909116	Met	N/A	64	f
Patient 32	x																Met	N/A	69	f
Patient 33	x																N/A	N/A	75	f
Patient 34	x																N/A	N/A	71	m
Patient 35	x																N/A	N/A	76	f
Patient 36	x																Unmet	N/A	73	m
Patient 37	x																Unmet	N/A	50	m
Patient 38	x																Met	N/A	60	f
Patient 39	x																N/A	N/A	77	m

Supplementary Table 2: Data and biosample use of patients with non-malignant intracranial disease or Conn's Syndrome

Identifier	CXCR4 PET/CT	Histology (IF)	Lightsheet	scRNA-Seq (CD45+) sc-cohort 1		Immuno-profiling	Medical center	Diagnosis	Age	Sex
				Cranial Bone	PBMCs					
Patient c1	x						University Hospital Essen	Conn's syndrome	53	f
Patient c2	x							Conn's syndrome	42	m
Patient c3		x	x					Subdural hematoma	77	m
Patient c4				x	x			Cavernous hemangioma	49	f
Patient c5				x	x	x		Subdural hematoma	65	m
Patient c6		x				x		Normal-pressure hydrocephalus	72	m
Patient c7		x						Subdural hematoma	50	m
Patient c8				x	x	x		Cerebral aneurysm	61	f
Patient c9				x	x	x		Cerebral aneurysm	47	f
Patient c10				x	x	x		Subdural hematoma	77	m
Patient c11	x							Conn's syndrome	44	m
Patient c12	x							Conn's syndrome	46	m
Patient c13	x							Conn's syndrome	67	m
Patient c14	x							Conn's syndrome	60	m
Patient c15		x						Cerebral aneurysm	55	m
Patient c16		x						Subdural hematoma	83	f
Patient c17		x						Subdural hematoma	80	m

Supplementary Table 3: Wilcoxon test data, related to Fig. 3i.

Activation:Effector function signature in all effector cells - summarized statistical results					
vs.	PBMC	Cranial Bone	Tumor	Ctrl PBMC	Ctrl Cranial Bone
PBMC		****	****	****	****
Cranial Bone	4,58E-53		****	****	****
Tumor	1,51E-10	3,04E-52		****	****
Ctrl PBMC	5,08E-31	5,14E-205	3,46E-113		****
Ctrl Cranial Bone	9,00E-39	4,96E-11	1,66E-23	1,53E-193	

Extracted statistical results

.y.	group1	group2	n1	n2	statistic	p	p.adj	p.adj.signif
value	PBMC	Cranial Bone	653	3304	666366,5	6,55E-54	4,58E-53	****
value	PBMC	Tumor	653	2300	627812	1,51E-10	1,51E-10	****
value	PBMC	Ctrl PBMC	653	1333	575690,5	1,27E-31	5,08E-31	****
value	PBMC	Ctrl Cranial Bone	653	2644	576905	1,80E-39	9,00E-39	****
value	Cranial Bone	Tumor	3304	2300	4712733	5,06E-53	3,04E-52	****
value	Cranial Bone	Ctrl PBMC	3304	1333	3465783,5	5,14E-206	5,14E-205	****
value	Cranial Bone	Ctrl Cranial Bone	3304	2644	4807143	2,48E-11	4,96E-11	****
value	Tumor	Ctrl PBMC	2300	1333	2224680,5	4,32E-114	3,46E-113	****
value	Tumor	Ctrl Cranial Bone	2300	2644	2535009,5	5,54E-24	1,66E-23	****
value	Ctrl PBMC	Ctrl Cranial Bone	1333	2644	745311,5	1,70E-194	1,53E-193	****

Captions for Supplementary Videos 1–4

Supplementary Video 1. 3D-View of maximum intensity projection of CXCR4 radiolabeling in patient with glioblastoma (Patient 2; Fig. 1c).

Supplementary Video 2. 3D-View of a whole mount immunofluorescence preparation of a cranial bone fragment of a patient with non-malignant intracerebral disease (Patient c3; Fig. 1f).

Supplementary Video 3. 3D-View of a whole mount immunofluorescence preparation of a cranial bone fragment of a patient with glioblastoma (Patient 7; Fig. 1h).

Supplementary Video 4. 3D-View of a whole mount immunofluorescence preparation of a cranial bone fragment of a patient with glioblastoma (Patient 6; Fig. 1i).

Extended Data Figure Legends

Extended Data Fig. 1: Clinical [^{68}Ga]Ga-Pentixafor radiolabeling. a, Representative imaging results of 19 patients with newly-diagnosed glioblastoma prior to neurosurgical tumor removal (patient 1 and 2 also included in Fig. 1). Clinical radiolabeling of CXCR4 and CT/MRI fusion allows identification of glioblastoma (asterisks) and surrounding encephalic and cranial structures. **b,** Representative Pentixafor PET-CT imaging data obtained from six patients diagnosed with Conn's syndrome, as a control, not suffering from intracranial neoplasia. Note the absence of tracer accumulation within the cranial bone.

Extended Data Fig. 2: Immune cell accumulation in the cranial bone of patients with glioblastoma. a, Confocal immunofluorescence imaging of large CB tissue sections from four additional patients with non-malignant intracranial disease (NTC, non-tumor control; patients c15, c3, c16, c17), complementing presentation in Fig. 1f,g. Scale bars indicated. **b,** CB histological appearance of samples from two additional patients with glioblastoma (patients 29, 30), complementing presentation in Fig. 1h-k. Magnifications in the insets, scale bars indicated. **c,** Graphs present estimated immune cell frequencies in CB large tissue sections, quantified by labeling with DAPI and CD45 (data from n =2 glioblastoma (GB), and n = 4 NTC counting cells in 12 vs. 20 cavities, respectively). **d,** Schematic illustrating derivation of vital cells from the CB cavities for follow-up investigation.

Extended Data Fig. 3: Immune cell derivation and cell type identification. a, Illustration of the workflow for sample processing and analysis of cells from sc-cohort 1 (see Methods). Samples from CB (n = 13), Tumor tissue (n = 6), and PBMC (n = 10) enriched by CD45$^+$ magnetic cell isolation. Single cells were further analyzed by scRNA-seq (10X Genomics) and integrated data were used for subsequent analyses. **b,** Split UMAP plots visualizing the distribution of annotated single cells by source. **c,** UMAP visualization of listed canonical marker genes of annotated cell types. Cells are colored by the respective gene set enrichment scores calculated via AUCell. **d,** Global UMAP of T cell types. Note, cells annotated as unknown or low quality were excluded from subsequent analyses. **e,** Bubble plot depicts the average expression levels and the fractions of cells expressing selected marker genes across the T cell types annotated in (d). **f,** Cluster-based annotation of CD8$^+$ T cell subspace.

Extended Data Fig. 4: Immune cell quantification based on the Immunoprofiling Assay. a, Representative gating strategy. Identified phenotypes, sample origin/number as indicated. **b,** Boxplot extend from 25th to 75th percentile, displaying median and minimum/maximum ranges as whiskers, summarizing frequency data (% of CD45$^+$ non-granulocytes) of indicated immune cell phenotypes, separated by source. Biological replicates, n indicated in (a). **c,** Representative dotplot displaying selected CD8$^+$ T cell phenotypes, as indicated in red. Note, analysis excluded naive CD8$^+$ T cells. **d,** Stacked bar plot indicating phenotype distribution per patient and source from listed patients. **e,** Graphs show frequencies of phenotypes in paired samples. Biological replicates (n=8). Two-tailed paired t-test; p values indicated.

Extended Data Fig. 5: Myeloid compartment. a, Phate plot representing the reference-based annotation of myeloid phenotypes in the single cell data presented in main Fig. 2. **b,** Phate plot as in (a), displaying the distribution of myeloid cells, color coded by source. **c,** Stacked barplots indicate frequencies of myeloid cells per source and disease condition. **d,** Cytometric profiling of myeloid cells. Gating strategy used to identify potential monocytic myeloid-derived suppressor cells (M-MDSCs) utilizing the listed markers. Note, raw data derived from assay shown in Extended Data Fig. 4. **e,** Boxplot extend from 25th to 75th percentile, displaying median and minimum/maximum ranges as whiskers, summarizing frequency data of potential M-MDSCs from (d), separated by source. Biological replicate data from (n) patients: GB-CB (8), -PBMC (8), -Tumor (7), -dBM (4); NTC-CB (5), -PBMC (4).

Extended Data Fig. 6: Distinct cytometric gating strategies for selected experimental approaches. a, related to main Fig. 3c, re-stimulation assay. Gating used to identify CD8$^+$ T cell subsets at different stages (CD45 expanded, re-stimulation I, II and III). **b,** related to main Fig. 4b, acutely isolated CD45$^+$ immune cells. Gating used to identify CD3$^+$ T cells expressing S1PR1. **c,** related to main Fig. 4c. Sub-characterization of S1PR1$^+$ CD8$^+$ T cell phenotypes from (b).

Extended Data Fig. 7: Developmental range assessment. a, Density plot illustrates the distribution of glioblastoma 3' GEX CD8$^+$ T cell data across the complete range of CytoTRACE scores, split by source. Note uniform distribution across all developmental stages in CB. **b,** Violin plot illustrating distribution of CBe CD8$^+$ T cells across CytoTRACE scores from (a), split by source. Boxplots display median, quartiles, and values within 1.5 * interquartile range as whiskers. Biological replicate data from (n) patients: Cranial Bone (5), Tumor (3). No statistically significant difference detected by two-sided Wilcoxon rank sum test with p value adjustments Holm method (SeuratExtend).

Extended Data Fig. 8: Arrangement of immune cells in the cranial bone. a, Immunofluorescent labeling of CD3 (red; T cells) and CD20 (green; B cells) in histological section from CB fragments of one patient with non-malignant intracranial disease (patient c6), and one patient with glioblastoma (patient 10). Nuclei were DAPI counterstained (blue). Note the lack of higher morphological organization of the tissue. Follicular arrangements, which are characteristic for matured tertiary lymphoid structures are not evident. Scale bars: 10 µm. **b,** Gene set enrichment score of a 12-chemokine reference TLS signature[30] does not indicate enrichment in the CB single cell data set. Scores were calculated via AUCell and depicted as UMAP, colored by score or as a violin plot, respectively, split by biological replicate data source. Cranial Bone (n=13), PBMC (n=10) and Tumor tissue (n=6). Boxplots display median, quartiles, and values within 1.5 * interquartile range as whiskers. Gene set enrichment scores are shown across all annotated cell types (upper panel) or in B and T cells (CD4$^+$/CD8$^+$/MAIT) alone (lower panel).

Extended Data Fig. 9: Sample preparation and annotation of sc-cohort 2. a, Sample processing and analysis workflow. Single cells from CB, tumor tissue and distal bone marrow (n = 3; patients 21, 22, 24) were enriched for $CD45^+/CD14^-$ cells by magnetic cell separation and further processed for scRNA-seq (10x Genomics) **b,** UMAP projection of integrated space. Inset colored according to the gene set enrichment score of canonical T cell marker genes, calculated via AUCell. **c,** UMAP of all T cells displaying annotated subtypes. Cells annotated as low quality were excluded from subsequent analyses. **d,** UMAP plot of $CD8^+$ T cell subset colored by source. **e,** Annotated $CD8^+$ phenotypes by label-transfer from $CD8^+$ T cells of sc-cohort 1 (see Fig. 3e) using singleCellNet.

Extended Data Fig. 10: Correlation of clinical and PET-CT/MRI data. a, Design of study. **b,** Data considered for univariate analyses. Note that low number of cases per group (n < 10) precludes multivariate analysis. Abbreviations: A, Two-sided Fisher's exact test; B, two-sided Student's t-test; C, Log-rank (Mantel-Cox) test. n, number of patients with glioblastoma.
