# Supplementary material for: Cranioencephalic functional lymphoid units in glioblastoma
Source: Nat Med. 2024 Jul 31;30(10):2947–56. doi: 10.1038/s41591-024-03152-x (PMC11485206; doi:10.1038/s41591-024-03152-x)
Supplement: Supplementary file 2 — Reporting Summary [file 41591_2024_3152_MOESM2_ESM.pdf]

nature portfolio

Corresponding author(s): Bjoern Scheffler

Last updated by author(s): Jun 13, 2024

Reporting Summary

Nature Portfolio wishes to improve the reproducibility of the work that we publish. This form provides structure for consistency and transparency in reporting. For further information on Nature Portfolio policies, see our Editorial Policies and the Editorial Policy Checklist.

Statistics

For all statistical analyses, confirm that the following items are present in the figure legend, table legend, main text, or Methods section.

n/a	Confirmed	
☐	☒	The exact sample size (*n*) for each experimental group/condition, given as a discrete number and unit of measurement
☐	☒	A statement on whether measurements were taken from distinct samples or whether the same sample was measured repeatedly
☐	☒	The statistical test(s) used AND whether they are one- or two-sided<br>*Only common tests should be described solely by name; describe more complex techniques in the Methods section.*
☒	☐	A description of all covariates tested
☐	☒	A description of any assumptions or corrections, such as tests of normality and adjustment for multiple comparisons
☐	☒	A full description of the statistical parameters including central tendency (e.g. means) or other basic estimates (e.g. regression coefficient) AND variation (e.g. standard deviation) or associated estimates of uncertainty (e.g. confidence intervals)
☐	☒	For null hypothesis testing, the test statistic (e.g. *F*, *t*, *r*) with confidence intervals, effect sizes, degrees of freedom and *P* value noted<br>*Give P values as exact values whenever suitable.*
☒	☐	For Bayesian analysis, information on the choice of priors and Markov chain Monte Carlo settings
☒	☐	For hierarchical and complex designs, identification of the appropriate level for tests and full reporting of outcomes
☒	☐	Estimates of effect sizes (e.g. Cohen's *d*, Pearson's *r*), indicating how they were calculated

Our web collection on statistics for biologists contains articles on many of the points above.

Software and code

Policy information about availability of computer code

Data collection	Syngo.Via software (Siemens Healthineers) version 9.03; Brainlab Imaging software iPlanNet (iPlanNet Cranial 3.0); Imaris (Bitplane), version 9.7.1, AID iSpot EliSpot Software (AID Autoimmun Diagnostika) version 7.0, SpectroFlo software (version 3.0.3; Cytek), FACS Diva v 8.0.1.1 software (BD Biosciences), Procreate version 5.3.3
Data analysis	CT/MRI fusion was performed using Syngo.Via (Siemens Healthineers) or Brainlab cranial navigation software (iPlanNet Cranial v3.0).<br>Bioinformatic and statistical data analysis of scData was performed in the R environment, version 4.2.0 and v.4.1<br>RNA-Sequencing results were analyzed using the 10x Genomics Cell Ranger 7.0.1 pipeline (3' GEX data) and the Cell Ranger 7.0.1 multi pipeline (5' GEX+V(D)J data). Data were analyzed with available packages: Seurat (v4.1.1 and v4.3.0), DoubletFinder (v.2.0.3), Harmony (v.0.1.1), SingleR (v.1.10.0), AUCell (v.1.18.1), Azimuth (v1.0.2), phateR (v1.0.7), scRepertoire (v.1.11.0), SeuratExtend (v.0.6.0), CytoTRACE (v.0.3.3), Monocle3 (v.1.3.1), ggplot2 (v.3.4.3), singleCellNet (v.0.1.0) and the Python package Palantir (v1.0.1). Tumor reactivity analysis was done using the predicTCR model under xgboost (v1.7.4).<br>ELISpot data were acquired using the AID EliSpot Software (version 7.0) and analyzed with Fiji Software (version 1.0). Flow cytometry and ELISpot statistical analyses were performed using Prism software (v 9.5.1 GraphPad) or Microsoft Excel (version 16.79.2). Univariate analysis of survival data was executed in SPSS (v.29.0.2.0).<br><br>Drawings by use of Procreate v5.3.3. Composition of figures in Affinity Designer v1.10.6.

For manuscripts utilizing custom algorithms or software that are central to the research but not yet described in published literature, software must be made available to editors and reviewers. We strongly encourage code deposition in a community repository (e.g. GitHub). See the Nature Portfolio guidelines for submitting code & software for further information.

Data

Policy information about availability of data

All manuscripts must include a data availability statement. This statement should provide the following information, where applicable:

- Accession codes, unique identifiers, or web links for publicly available datasets
- A description of any restrictions on data availability
- For clinical datasets or third party data, please ensure that the statement adheres to our policy

scRNA-seq data were deposited into NCBI Gene Expression Omnibus (GEO) and are available under accession # GSE233304. The hg38 human reference genome and the human V(D)J reference (GRCh38) are available under https://www.10xgenomics.com/support/software/cell-ranger/latest/release-notes/cr-reference-release-notes#cr7-0. All other data and materials are available in the manuscript and supplementary data.

Research involving human participants, their data, or biological material

Policy information about studies with human participants or human data. See also policy information about sex, gender (identity/presentation), and sexual orientation and race, ethnicity and racism.

Reporting on sex and gender	Sex/gender was not considered in the study design.
Reporting on race, ethnicity, or other socially relevant groupings	Race, ethnicity or other socially relevant groupings were not considered in the study design.
Population characteristics	30 patients (age 45-83) with newly-diagnosed IDH-WT Glioblastoma CNS Grade 4;<br>9 additional patients (age 50-77) diagnosed with Glioblastoma for retrospective analysis (Reference PMID: 26909116);<br>11 patients (age 49-83) presenting with non-malignant intracranial disease and 6 patients (age 42-67) presenting with Conn's syndrome for PET/CT analysis, their biological material and/or data was used as a control<br>Detailed populations characteristics are outlined in supplementary table 1 and 2.
Recruitment	All patients with suspected glioblastoma scheduled for surgical tumor removal at the University Hospital Essen were recruited by a physician. There was no specific bias in patient selection. Informed consent was obtained from every eligible patient prior to their inclusion in the study. PET-CT data of patients who received Pentixafor were included following confirmation of the neuropathological diagnosis "Glioblastoma IDH-WT CNS WHO grade 4". All consecutive patients who had sufficient material obtained from surgical specimens and a confirmed glioblastoma diagnosis were further included in the molecular studies.<br>Patients presenting with non-malignant intracranial diseases were recruited similarly. All consecutive patients who had sufficient material obtained from surgical specimens and received a diagnostic confirmation of a non-malignant intracranial disease were included. Additionally, patients with Conn's syndrome who underwent an in-house PET/CT with Pentixafor and had a complete head scan available were included in the study.<br><br>Furthermore, PET-CT/MRI data from nine patients from the University Hospital Würzburg were retrospectively compiledfrom published data (Reference PMID: 26909116). Data from patients diagnosed with glioblastoma and who had undergone a CXCR4-PET/CT with Pentixafor in the original study were evaluated.<br><br>Correlation of clinical and PET-CT/MRI data was performed for all patients who received Pentixafor (both prospective and retrospective recruitment), were treated by surgery and radio- and/or temozolomide-based chemotherapy subsequent to CXCR4-PET/CT, had available survival data with an overall survival of at least three months and had follow-up MRI data available (see study design in Extended Data Fig. 10a).
Ethics oversight	Written informed consent was obtained from all participants of this study; all procedures were performed in accordance with the Declaration of Helsinki and approved by the local ethics committees (University Hospital Essen approval #19-8706-BO, #22-10564-BO; University Hospital Würzburg approval #20230824 01).

Note that full information on the approval of the study protocol must also be provided in the manuscript.

Field-specific reporting

Please select the one below that is the best fit for your research. If you are not sure, read the appropriate sections before making your selection.

☒ Life sciences ☐ Behavioural & social sciences ☐ Ecological, evolutionary & environmental sciences

For a reference copy of the document with all sections, see nature.com/documents/nr-reporting-summary-flat.pdf

Life sciences study design

All studies must disclose on these points even when the disclosure is negative.

Sample size	No sample size was predetermined before the study. For patient-derived samples, all consecutive samples with sufficient material were

Sample size	included in the study.
Data exclusions	Patients were excluded from survival analysis if they did not meet analysis inclusion criteria (treated by surgery and radio- and/or temozolomide-based chemotherapy subsequent to CXCR4-PET/CT, available survival data with an overall survival of at least three months and follow-up MRI data available, see "Recruitment" and Extended Data Fig. 10a). All patients of whom adequate biological material could be derived were included in the study.
Replication	- We replicated CXCR4 PET-CT findings by collecting individual datasets from 19 patients with glioblastoma and 6 patients with Conn's syndrome as a control.<br>-We replicated single cell RNA seq findings by collecting data from independent samples of 11 patients with glioblastoma and 5 patients with non-malignant intracranial disease. Biological replicates of individual samples from patients with glioblastoma were included: 3' GEX - 3x PBMC, 5x CB, 3x Tumor; 5'GEX+V(D)J - 6x 2x PBMC, 6x CB, 6x Tumor, 3x dBM. Biological replicates of individual samples from patients with non-malignant intracranial disease were included: 3' GEX - 5x PBMC, 5x CB.<br>- Replication of lightsheet and histology data involved analysis of bone material from 7 patients with glioblastoma and 6 patients with non-malignant intracranial disease.<br>- Functional in-vitro studies (ELISpot) were conducted on primary cells derived from 9 patients with glioblastoma (biological replicates) and were conducted in duplicates and triplicates (technical replicates) as indicated in the respective figure legends.<br>- T cell activation monitored by T cell clustering/aggregation was conducted on primary cells of one glioblastoma patient in triplicates per source (as indicated in the respective graph).<br>- Resilience assays were conducted on primary cells derived from 6 patients with glioblastoma (biological replicates) and was performed in triplicates per patient and source (as indicated in the figure, legend, and in the methods section)<br>- Immunoprofiling conducted on freshly isolated cell samples derived from 12 patients with glioblastoma and 5 patients with non-malignant intracranial disease (biological replicates). Biological replicates of individual samples from patients with glioblastoma were included: 8x CB, 8x PBMC, 7x Tumor, 4x dBM. Biological replicates of individual samples from patients with non-malignant intracranial disease were included: 5x CB, 4x PBMC (cytometric profiles of one PBMC sample could not be collected due to insufficient input cell numbers).<br>- S1PR1 levels were determined by flow cytometry on biological replicates, as indicated in the figure and in the corresponding figure legends (5x PBMC + 5x dBM and 7x PBMC + 7x CB)<br><br>With the exception of failed cytometric immunoprofile (one PBMC sample as stated), all attempts of replication were successful.<br>Further details on replication can be found in the supplementary tables 1 and 2, and in the respective figure or figure legend
Randomization	Experiments were not randomized.
Blinding	With the exception of PET data correlation with patient survival (Fig. 4m-o and Extended Data Fig. 10), the investigators were not blinded to allocation and the conditions of the experiments.

Reporting for specific materials, systems and methods

We require information from authors about some types of materials, experimental systems and methods used in many studies. Here, indicate whether each material, system or method listed is relevant to your study. If you are not sure if a list item applies to your research, read the appropriate section before selecting a response.

Materials & experimental systems

n/a	Involved in the study	
☐	☒	Antibodies
☐	☒	Eukaryotic cell lines
☒	☐	Palaeontology and archaeology
☒	☐	Animals and other organisms
☒	☐	Clinical data
☒	☐	Dual use research of concern
☒	☐	Plants

Methods

n/a	Involved in the study	
☒	☐	ChIP-seq
☐	☒	Flow cytometry
☐	☒	MRI-based neuroimaging

Antibodies

Antibodies used	PE/Dazzle(TM) 594 anti-human CD45 antibody, Clone HI30, BioLegend Cat# 304051, RRID:AB_2563567<br>Alexa Fluor(R) 647 anti-human CD34 antibody, Clone 581, BioLegend Cat# 343508, RRID:AB_1877133<br>PE/Dazzle(TM) 594 anti-human CD3 antibody, Clone UCHT1, BioLegend Cat# 300450, RRID:AB_2563618,<br>CD20 Monoclonal Antibody (L26), Alexa Fluor 488, eBioscience, Thermo Fisher Scientific Cat# 53-0202-80, RRID:AB_10734357<br>Human Fc Block, BD Pharmigen, Cat# 564220, RRID: AB_2869554<br>BV421 CD95 antibody, Biolegend Cat# 305623, RRID: AB_2561830<br>BV786 CD45RA antibody, BD Bioscience Cat# 563870, RRID: AB_2738459<br>BV711 CD8 antibody, Biolegend Cat# 344733, RRID: AB_2565242<br>BV510 CCR7 antibody, Biolegend Cat# 353231, RRID: AB_2561937<br>APC CD4 antibody, BioLegend Cat# 317415, RRID: AB_571944<br>PE CD56 antibody, BD Bioscience Cat# 555516, RRID: AB_395906<br>FITC CD161 antibody, Biolegend Cat# 339905, RRID: AB_2564140<br>PeCy7 CD3 antibody, BioLegend Cat# 344815, RRID: AB_10641705<br>BV650 PD-1 antibody, Biolegend Cat# 329949, RRID: AB_2566361<br>eFluor 660 S1PR1 antibody, Thermo Scientific, Cat# 50-3639-42, RRID: AB_2574208

eFluor 660 Isotype Control IgG1, κ, Thermo Scientific, Cat# 50-4714-82, RRID: AB_10597301
PE CD4 antibody, Biolegend Cat# 317410, RRID: AB_571954
Rat anti-CD45, Bio-Rad, Cat# MCA345G, RRID: AB_770081
Goat anti-CD146, R and D Systems, Cat# AF932, RRID: AB_355721
Rabbit anti-CXCR4, Thermo Fisher Scientific, Cat# PA3-305, RRID: AB_2091817
Cytek® 25-Color Immunoprofiling Assay, cFluor® Reagent Kit (18C), Cytek; Cat# R7-40002
Immunoprofiling Kit, 7 Color (Brilliant Violet™), BioLegend (acc. to Cytek protocol); Item#: 900004160
Ultra-LEAF(TM) Purified anti-human HLA-DR, BioLegend, Cat# 307648, RRID: AB_2561493
Ultra-LEAF(TM) Purified anti-human HLA-A,B,C, BioLegend, Cat# 311428, RRID:AB_2561492

Validation

All antibodies were commercially available and were validated by the manufacturer
-BioLegend: https://www.biolegend.com/de-de/quality/quality-control
Info BioLegend: "All of our products undergo industry-leading rigorous quality control (QC) testing to ensure the highest level of performance and reproducible results. Each lot is compared to an internally established “gold standard” to maintain lot-to-lot consistency." Antibodies are tested on different cells/tissues, expression levels and applications to ensure sensitivity and specificity,

-BD: https://www.bdbiosciences.com/en-eu/products/reagents/flow-cytometry-reagents/research-reagents/quality-and-reproducibility
Info BD: "The specificity is confirmed by using multiple applications that may include a combination of flow cytometry, immunofluorescence, immunohistochemistry or western blot to test a combination of primary cells, cell lines or transfectant models." Similar to Biolegend, antibodies are tested on a combination of cells/models and multiple applications to ensure specificity.

-Bio-Rad: https://www.bio-rad-antibodies.com/our-guarantees-to-you.html
Info Bio-Rad: "We perform both in house testing and a thorough review of published literature, to determine which applications are suitable for use with each of our antibodies." Antibodies are tested thoroughly and Bio-Rad holds the ISO 9001: 2015 certification as an internationally recognized standard.

-R&D systems: https://www.rndsystems.com/products/rd-systems-approach-antibody-quality
Info R&D systems: "R&D Systems carefully tests every antibody we produce to ensure outstanding performance. All antibodies are tested for cross-reactivity with closely related molecules using a variety of applications, including direct ELISA, to ensure specificity. These efforts are facilitated by our extensive library of in-house developed antigens." All antibodies are tested thoroughly undergoing multiple steps to ensure specificity of each antibody.

-Thermo Fisher Scientific: https://www.thermofisher.com/de/de/home/life-science/antibodies/invitrogen-antibody-validation.html
Thermo Fisher Scientific Statement: "Expanded specificity testing methods to ensure superior antibody results." Thermo Fisher Scientific adheres to the recommendations of the International Working Group for Antibody Validation (IWGAV) to ensure antibody specificity.

-Cytek: Cytek® 25-Color Immunoprofiling Assay, cFluor® Reagent Kit (18C)+ Immunoprofiling Kit, 7 Color (Brilliant Violet™), BioLegend:
"The 25-color assay has been optimized and titrated for use with 7 Brilliant Violet™ reagents available from Biolegend® for analyzing human PBMC and whole blood on Cytek’s Aurora systems equipped with violet, blue, yellow-green, and red lasers."
Immunoprofiling Kits were obtained from and validated by Cytek and BioLegend, see Cat# above. The use of the suggested antibody combination has been validated, a full validation report for the immunoprofiling kit can be found via https://cytekbio.com, Cat# R7-40002

Further available references:
Antigen;Clone;Company; RRID; Reference
CD45; HI30; BioLegend; RRID:AB_2563567; Knapp W, et al. 1989. Leucocyte Typing IV. Oxford University Press. New York./Rees LE, et al. 2003. Clin. Exp. Immunol. 134:497 (IF/IHC)
CD34; 581; BioLegend; RRID:AB_1877133; Schlossman SF, et al. 1995. Leukocyte Typing V:White Cell Differentiation Antigen. New York:Oxford University Press./Umland O, et al. 2003. J. Histochem. Cytochem. 51:977 (IF/IHC)
CD3; UCHT1; BioLegend; RRID:AB_2563618; Ryschich E, et al. 2003. Tissue Antigens 62:48./Mack CL, et al. 2004. Pediatr. Res. 56:79. (IF/IHC)
CD20; L26; Thermo Fisher Scientific; RRID:AB_10734357; Moysi E et al. 2021 Front Immunol. 9;12:683396./Rashid R et al. Sci Data. 2019 Dec 17;6(1):323. (IF/IHC)
Human Fc Block, Fc1, BD Pharmigen, RRID: AB_2869554, FC routinely tested by the manufacturer.
CD95; DX2; BioLegend; RRID: AB_2561830; Kishimoto T, et al. Eds. 1997. Leucocyte Typing VI. Garland Publishing Inc. New York./Xie S, et al. 2010. J. Immunol. 184:2289. (FC)/ FC - Quality tested by Biolegend
CD45RA; HI100, BD Biosciences; RRID: AB_2738459; Knapp W. et al., ed. Leucocyte typing IV : white cell differentiation antigens. Oxford New York: Oxford University Press; 1989:1-1182./ Koristka S, et al . J Immunol. 2012; 188(3):1551-8. (FC, FC sorting) /FC routinely tested by the manufacturer.
CD8; SK1; BioLegend; RRID: AB_2565242; Montel-Hagen A et al. 2019. Cell stem cell. 24(3):376-389 (FC) / FC - Quality tested by Biolegend
CCR7; G043H7; BioLegend; RRID: AB_2561937; Martin AR, et al. 2020. EBioMedicine. 62:103102 / FC - Quality tested by Biolegend
CD4; OKT4; BioLegend; RRID: AB_571944; Knapp W, et al. 1989. Leucocyte Typing IV. Oxford University Press. New York./Kmieciak M, et al. 2009. J. Transl. Med. 7:89. (FC)/ FC - Quality tested by Biolegend
CD56; B159; BD Biosciences; RRID: AB_395906; Maraiani E, et al., ed. Leucocyte typing V : white cell differentiation antigens : proceedings of the fifth international workshop and conference held in Boston, USA, 3-7 November, 1993. Oxford: Oxford University Press; 1995:1394-1397./Crotta S et al J Exp Med. 2002; 195(1):35-41. (FC) / FC routinely tested by the manufacturer.
CD161; HP-3G10; BioLegend; RRID: AB_395906; Maluski M, et al. 2019. J Clin Invest. 129:5108./ FC - Quality tested by Biolegend
CD3; SK7; BioLegend; RRID: AB_10641705; Kan EA, et al. 1983. J. Immunol. 131:536./Alfonso-Dunn R, et al. 2022. Front Immunol. 13:926318/FC - Quality tested by Biolegend
PD-1; EH12.2H7; BioLegend; RRID: AB_2566361; Radziewicz H, et al. 2010. J. Immunol. 184:2410./Conrad J, et al. 2011. J. Immunol. 186:6871. / FC - Quality tested by Biolegend
S1PR1; SW4GYPP; Thermo Scientific; RRID: AB_2574208; Cantero-Pérez J, et al. 2019. Nat. Commun 18;10(1):4739. (FC) / FC as listed

application from Thermo Fisher Scientific
eFluor 660 Isotype Control IgG1, κ, P3.6.2.8.1, Thermo Scientific; Manufacturer info: Isotype Control has been tested by FC, can be used at the same concentration as the experimental antibody.
CD4; OKT4; Biolegend; RRID: AB_571954; Knapp W, et al. 1989. Leucocyte Typing IV. Oxford University Press. New York. / Xie G et al. Cell Rep. 2021 Apr 27;35(4):109038. (FC) / FC - Quality tested by Biolegend
CD45; YTH14.5; Bio-Rad; RRID: AB_770081; Bindon, C.I. et al. (1985) Therapeutic potential of monoclonal antibodies to the leukocyte-common antigen. Synergy and interference in complement-mediated lysis. Transplantation. 40 (5): 538-44. Established in-house for the application in quantitative multiplex immunofluorescence imaging at a dilution of 1:25 (4°C overnight)
CD146; Polyclonal; R and D Systems, RRID: AB_355721; Keuschnigg J, et al. PLoS One. 2013 Sep 10;8(9):e74293. (IF)
CXCR4; Polyclonal; Thermo Fisher Scientific; RRID: AB_2091817; Pluchino N, et al. J Cell Mol Med. 2020 Feb;24(4):2464-2474 (IF) / IF as listed application from Thermo Fisher Scientific
anti-human HLA-DR; L243; BioLegend; RRID: AB_2561493; Wang RF, et al. 1999. Science 284:1351 (Block)
anti-human HLA-A,B,C; W6/32; BioLegend; RRID:AB_2561492; Dangaj D, et al. Cancer Cell. 2019 Jun 10;35(6):885-900.e10. (Block)

Eukaryotic cell lines

Policy information about cell lines and Sex and Gender in Research

Cell line source(s)	No commercially available cell line was used in this study.
Authentication	No authentication was performed.
Mycoplasma contamination	Mycoplasma contamination is routinely assessed in our lab. No mycoplasma contamination was recorded during experiments reported in manuscript.
Commonly misidentified lines (See ICLAC register)	n/a, see above.

Flow Cytometry

Plots

Confirm that:

☒ The axis labels state the marker and fluorochrome used (e.g. CD4-FITC).

☒ The axis scales are clearly visible. Include numbers along axes only for bottom left plot of group (a 'group' is an analysis of identical markers).

☒ All plots are contour plots with outliers or pseudocolor plots.

☒ A numerical value for number of cells or percentage (with statistics) is provided.

Methodology

Sample preparation	Corresponding methods sections: Tumor tissue processing, Bone sample processing, Blood sample and dBM processing, Selection and preservation of immune cells and Flow cytometry
Instrument	Cytek Aurora flow cytometer configured in the 5L setup (16UV-16V-14B-10YG-8R),<br>FACS Celesta flow cytometer (BD Biosciences)
Software	Immunoprofiling: spectral profiles were acquired using SpectroFlo software (version 3.0.3; Cytek). Alternative gating strategy for the identification of potential M-MDSCs was carried out via FlowJo, version 10.9.0.<br>All other flow cytometry measurements were acquired and analyzed with the FACS Diva v 8.0.1.1 software (BD Biosciences) and FlowJo, version 10.9.0.
Cell population abundance	Events of each gate were exported and cell subset frequencies were calculated relative to the amount of cells in a respective reference gate, as stated on the y-axis of each plot. Calculation was done in Microsoft excel, statistical analyses were executed in graphpad prism.
Gating strategy	Immunoprofiling (Cytek): Unmixing was performed using the manufacturer's recommended reference controls with autofluorescence extraction enabled. SSC-H/SSC-B-H plots were used to determine Leukocytes, followed by FSC-A/FSC-H plots to determine singlet gates. Stable flow (FSC-A/Time(min) plot) and viability (SSC-A/ViaDye Red) was ensured and distinct cell populations were quantified using an enhanced gating strategy based on Cytek's recommendations for the immunoprofiling kit. Gating strategies are outlined in Extended Data Fig. 4 and 5.<br><br>All other flow cytometry measurements: SSC-A/FSC-A plots were used to determine cells of interest and gate out debris, followed by FSC-A/FSC-H plots to determine singlet gates. Viability was ensured using 7AAD/FSC-H and distinct cell populations were identified. Gating strategy of human CD8+ T cell subsets was based on the "Guidelines for the use of flow cytometry and cell sorting in immunological studies (third edition) (Cossarizza A et al Eur J Immunol. 2021 Dec;51(12):2708-3145. PMID: 34910301.). Isotype controls were utilized to indicate the boundaries between positive and negative populations. Gating strategies are outlined in Extended Data Fig. 6

☒ Tick this box to confirm that a figure exemplifying the gating strategy is provided in the Supplementary Information.

Magnetic resonance imaging

Experimental design

Design type	MRI with contrast enhancement as part of clinical routine, used for secondary fusion with PET-CT imaging data.
Design specifications	MRI with contrast enhancement as part of clinical routine
Behavioral performance measures	n/a

Acquisition

Imaging type(s)	Structural MRI
Field strength	3 Tesla
Sequence & imaging parameters	Standard parameters of the radiology departments of the institution.
Area of acquisition	Whole Brain

Diffusion MRI ☐ Used ☒ Not used

Preprocessing

Preprocessing software	No preprocessing was performed; MR images were directly used for used for secondary fusion with PET-CT imaging data (Syngo.Via software)
Normalization	n/a as no preprocessing was performed
Normalization template	n/a as no preprocessing was performed
Noise and artifact removal	n/a as no preprocessing was performed
Volume censoring	n/a as no preprocessing was performed

Statistical modeling & inference

Model type and settings	No statistical modeling and inference performed
Effect(s) tested	n/a

Specify type of analysis: ☒ Whole brain ☐ ROI-based ☐ Both

Statistic type for inference (See Eklund et al. 2016)	n/a
Correction	n/a

Models & analysis

n/a	Involved in the study
☒	☐ Functional and/or effective connectivity
☒	☐ Graph analysis
☒	☐ Multivariate modeling or predictive analysis
